# On the Existence of XOR-Based Codes for Private Information Retrieval with Private Side Information

**DOI:** 10.3390/e23101287

**Published:** 2021-09-30

**Authors:** Murali Krishnan K. H., Jagadeesh Harshan

**Affiliations:** 1 Bharti School of Telecom Technology and Management, Indian Institute of Technology Delhi, New Delhi 110016, India; 2 Department of Electrical Engineering, Indian Institute of Technology Delhi, New Delhi 110016, India; jharshan@ee.iitd.ac.in

**Keywords:** private information retrieval, joint privacy, privacy with side information, XOR-based codes

## Abstract

We consider the problem of Private Information Retrieval with Private Side Information (PIR-PSI), wherein the privacy of the demand and the side information are jointly preserved. Although the capacity of the PIR-PSI setting is known, we observe that the underlying capacity-achieving code construction uses Maximum Distance Separable (MDS) codes therefore contributing to high computational complexity when retrieving the demand. Pointing at this drawback of MDS-based PIR-PSI codes, we propose XOR-based PIR-PSI codes for a simple yet non-trivial setting of two non-colluding databases and two side information files at the user. Although our codes offer substantial reduction in complexity when compared to MDS-based codes, the code-rate marginally falls short of the capacity of the PIR-PSI setting. Nevertheless, we show that our code-rate is strictly higher than that of XOR-based codes for PIR with no side information. As a result, our codes can be useful when privately downloading a file especially after having downloaded a few other messages privately from the same database at an earlier time-instant.

## 1. Introduction

Private Information Retrieval (PIR) deals with the design of queries to a database to provide privacy to the messages downloaded by the user. A trivial way of achieving information-theoretic privacy is to download all the messages stored in the database so that the identity of the demand, i.e., the message the user wants, will be unknown. However, it is well known that this increases the download cost substantially. Ever since the problem of PIR was first introduced in [1], various methods have been introduced to efficiently retrieve the demand with privacy, including the code constructions that achieve the capacity of the classic PIR problem [2]. Other than the capacity-achieving code constructions, several low-complexity constructions are also known [3,4] for the classic PIR problem.

Since the contribution of [2], numerous variants of the PIR problem have gained attention [5,6,7]. Among them, an important variant is the problem of PIR with side information. In this setting, the user already knows one or more messages in the database, and she uses this information to reduce the download cost compared to that without side information. The work on PIR with side information began with the cache-aided PIR in [8]. Other crucial works on PIR-SI were followed in [9,10,11,12,13]. A prominent variant of PIR with side information is the problem of PIR with private side information (PIR-PSI), wherein the privacy of both the demand and the side information are jointly preserved, i.e., the database should not know which message is being queried by the user and which side information is available at her. The application of PIR-PSI has practical significance when users must query a message privately from a database after having downloaded a few other messages privately from the same database at an earlier time-instant. Suppose a user has already queried messages *A* and *B* with full privacy. Furthermore, suppose that the same user wants to query another message *C* at a later point in time. One way to query *C* is using the capacity-achieving fully private scheme [2]. Alternatively, the user can reduce the download cost for retrieving *C* using *A* and *B* as the side information. This way, as the number of side information messages increases, the download cost for a particular demand can potentially decrease. For these applications, PIR-PSI protocol ensures that demand is downloaded with the help of side information, while jointly preserving the privacy of both the demand and the side information.

### 1.1. Existing Codes for PIR-PSI Setting and Their Limitations

A solution for PIR-PSI was first developed by Kadhe et al. in [14] for the single database setting. Chen et al. further extended it in [15] for multiple databases in colluding and non-colluding environments. Both their schemes achieved capacity; however, both the schemes for single database and multiple databases mentioned in [14,15], respectively, rely on Maximum Distance Separable (MDS) codes. From the viewpoint of implementation, it is well known that any MDS-coded scheme would have high computational complexity compared to a counterpart code that is constructed using XOR bit additions. For example, consider the code in [15] for two non-colluding databases with three messages A,B,C with a length of eight bits per message. Let *A* be the demand and *C* be the side information for a particular user. As mentioned in [Section 4.1.1] in [15], the query would initially be in the form of Sun-Jafar’s capacity-achieving scheme mentioned in [2], with four bits of *A* retrieved out of seven bits queried from a database. This query of seven bits would be converted to an (n=13,k=7) systematic MDS code, e.g., a Reed-Solomon code, and the six bits of the non-systematic part would be downloaded. With the help of the side information *C*, the user can retrieve four demand bits from these six bits, therefore improving the rate. Here, the encoding operation for Reed-Solomon code would require 91 finite field multiplications and 78 finite field additions, which in turn are computation-intensive tasks compared to executing four XOR bit additions in Sun-Jafar’s capacity-achieving scheme. In general, any MDS code for PIR-PSI would depend on the message length *L*, as seen above, and *L* itself depends on $N^{K}$ (where *N* is the number of databases and *K* is the number of messages). Therefore, the computational complexity of MDS-coded PIR-PSI codes will exponentially increase as the number of messages increases. Please note that this discussion on complexity is not related to sub-packetization of the messages, instead it is only on the arithmetic operations post sub-packetization process.

### 1.2. Motivation

We note that a majority of existing PIR schemes such as in [2,7,16,17,18] are XOR-based code constructions, i.e., each database returns a set of XOR combinations of bits of various files, and similarly, the user also performs corresponding XOR operations to recover the message in demand. However, when tackling the PIR-PSI problems, instead of constructing XOR-based codes, MDS code-based constructions have been presented in [14,15]. Although these MDS codes achieve the capacity of the PIR-PSI setting, from a practical viewpoint, for a large number of messages, the number of computations would become prohibitively large to create a huge latency in downloading the demand. For instance, even though a computationally powerful database may return the queries to the user after finite field computations, performing similar finite field computations on this query for recovering the message at the user is challenging if the user’s device is not computationally powerful [19,20,21,22]. Therefore, existing PIR-PSI codes are not amenable to implementation in such cases. On the other hand, using the PIR scheme of [2], which is an XOR-based code construction, is not a good idea as it would affect the rate for not exploiting the side information. With this background, we ask the question: are there PIR-PSI codes based on XOR computations with rates strictly more than the code in [2]?

### 1.3. Contributions

**Figure 1:**
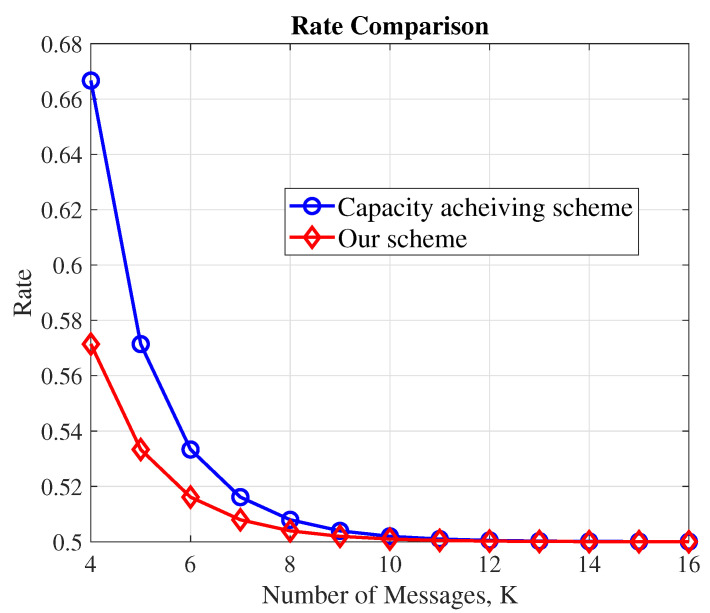
Rate of the proposed code construction along with the capacity of the PIR-PSI scheme for M=2 and N=2.

Inspired by the problem statement discussed above, we make the following contributions in this paper:

- We present the first XOR-based code construction for the PIR-PSI problem. Our code construction is applicable for the scenario when *K* messages, each of length $L=2^{K-1}$ bits, are replicated across N=2 non-colluding databases, and the user wishes to retrieve a message with M=2 side information at her side. Although the setting of N=2 databases and M=2 side information messages may be applicable to a specific distributed storage model, we highlight that the code contribution is non-trivial as it affirmatively answers the question posed in the motivation section, and moreover it is applicable for any *K*.
- Owing to the use of XOR-based queries, we show that our codes offer substantial reduction in the decoding complexity compared to the MDS-based counterparts of [15]. However, the rate of our codes marginally fall short of the capacity of the PIR-PSI problem for the setting of N=2 non-colluding databases with M=2 side information at the user (as seen in Figure 1). Nevertheless, the rates of our codes are strictly higher than that of the fully private codes [2] therefore advertising themselves as a preferred choice when privately downloading a message after having downloaded a few other messages privately from the same database at an earlier time-instant. For N=2 and M=2, a comparison between existing solutions for PIR-PSI and our solution is captured in Table 1.
- For the proposed code construction, we prove the joint-privacy property by explicitly showing that the query to one of the databases can be fixed, and then the query to the other database can be modified in such a way that any combination of side information can be used to retrieve any demand from the two databases. This is a novel approach to physically verify the privacy of any XOR-based PIR codes.

We believe that our work opens up a wide range of new problems for investigation, such as: (i) Does the XOR constraint in the code construction reduce the rate of the codes from the capacity of PIR-PSI? (ii) Are there capacity-achieving XOR-based code construction for PIR-PSI?, if not, what is the capacity of XOR-based PIR-PSI codes?

### 1.4. A Sneak-Peek at Our Codes

Before formally stating the problem statement, and presenting the code construction, we present an example for our codes with K=4. This example serves two purposes: (i) assists the reader to verify the PIR-PSI property explicitly, and (ii) confirms that the rate is higher than the code in [2]. Consider two databases $N_{1}$ and $N_{2}$ with four messages A,B,C and *D* of length eight bits each. Let a user want to retrieve message *A* with *B* and *C* as the side information. Table 2 shows the query obtained as per the proposed code construction to retrieve eight bits of *A* from using *B* and *C* as side information. In this description of the code, the notation $A_{1},A_{2},\ldots ,A_{8}$ denote the eight bits of the file *A*. Similarly, $\left\{B_{i}\;|\;1\leq i\leq 8\right\}$, $\left\{C_{i}\;|\;1\leq i\leq 8\right\}$, and $\left\{D_{i}\;|\;1\leq i\leq 8\right\}$ denote the eight bits of the files B,C and *D*, respectively.

To show the privacy of the code in Table 2, we fix the query to $N_{1}$ (that was generated when *A* is the demand and B,C are the side information), and then generate all possible queries to $N_{2}$ such that any demand can be retrieved using any two side information. All possible queries to $N_{2}$ when *D* is a side information, a byproduct, and the demand, are listed in Table 3, Table 4 and Table 5, respectively. In this context, a byproduct is a message that is neither side information nor demand. In all the three tables, the three-tuple format on the top row indicates (Demand, Side Information, Byproduct) combination for the query in that column. In each of the cases, we highlight that the structure of the queries to $N_{2}$ are unaltered compared to the original query to $N_{2}$ in Table 2.

From Table 3, Table 4 and Table 5, it is evident that from the perspective of $N_{1}$ the query given in Table 2 can be for any demand-side information combination. Since the structure of query at $N_{2}$ is exactly same as that of $N_{1}$, a similar reasoning can also be applied to show that for a query at $N_{2}$, any demand can be retrieved using any side information from $N_{1}$. Thus, $N_{1}$ and $N_{2}$ cannot identify the identity of the demand and the side information jointly, and hence, the scheme is jointly private.

The rest of the paper concerns the following sections. Section 2 presents the problem statement and related notations. Section 3 presents the code construction, whereas Section 4 provides the proof for joint privacy of the XOR-based codes for arbitrary values of *K*. Section 5 exemplifies the privacy proof for the case when K=7. Finally, some directions for future research are presented in Section 6. A preliminary version of this work is also available at [23].

## 2. Problem Statement

Consider two replicated non-colluding databases, $N_{1}$ and $N_{2}$ with *K* messages namely $X_{1},X_{2},\ldots ,X_{K-1},X_{K}$. All the *K* messages are of size $L=2^{K-1}$ bits which are independent and uniformly distributed. Therefore, we denote the *i*-th file $X_{i}$ as $X_{i}=\left[X_{i,1},X_{i,2},\ldots ,X_{i,L}\right]$. Among the *K* messages, let $X_{\gamma }$, for γ∈[K], be the demand message for a user who already has M=2 side information messages $X_{\alpha }$ and $X_{\beta }$, such that α,β∈[K]\{γ} satisfying α≠β. In this model the messages other than $X_{\gamma },X_{\alpha }$ and $X_{\beta }$ are referred to as byproducts. The user wishes to leverage the knowledge of $X_{\alpha }$ and $X_{\beta }$ to retrieve $X_{\gamma }$ by downloading fewer bits from each database compared to retrieving $X_{\gamma }$ using the scheme in [2] without using the side information. At the same time, the user also wants to jointly preserve the privacy of the demand $X_{\gamma }$ and the side information messages $X_{\alpha }$ and $X_{\beta }$, i.e., the indices γ, α and β should be unknown to both databases. In the context of this work, the code for retrieving $X_{\gamma }$ should be such that each database provides the user a set of XOR additions of the bits of the *K* messages. This way, the user would be able to retrieve $X_{\gamma }$ by performing simple XOR bit additions on the downloaded bits while using the prior knowledge of $X_{\alpha }$ and $X_{\beta }$.

Towards constructing an XOR-based code for PIR-PSI, we make the following definitions. In order to retrieve the demand $X_{\gamma }$ using the side information $X_{\alpha }$ and $X_{\beta }$, every combination of message bits submitted to a database is referred to as *codeword*, the set of codewords submitted to a database is called a *query*, and the union of queries submitted to $N_{1}$ and $N_{2}$ is referred to as the *code*. When designing the codewords of a query it is important to find the right set of XOR combinations of the *K* message indices, and then choose the appropriate bit locations of the message indices in the XOR combinations. To formally describe the XOR combinations of the message indices, we introduce the following definitions.

**Definition** **1.** *For a finite set $M=\{M_{1},M_{2},\ldots ,M_{P}\}$ containing Z distinct variables, we define a mapping *Φ* such that $\Phi \left(M\right)=\sum_{i=1}^{Z}M_{i}$ if Z≥1, and Φ(M)=ϕ if M is empty set.*

**Definition** **2.** *With $X=\{X_{1},X_{2},\ldots ,X_{K}\}$, we define a power set-based skeleton structure P(X)={Φ(V)|∀V∈PS(X)}, where Φ(·) is as introduced in Definition 1, and PS(X) is the power set of X.*

From Definition 2, it is clear that the queries (without specifying the bit locations of each message) submitted to $N_{1}$ and $N_{2}$ must be subsets of P(X) \{ϕ}, wherein the + operator in Φ is treated as XOR operation. Henceforth, throughout the paper, the queries submitted to $N_{1}$ and $N_{2}$ to retrieve $X_{\gamma }$ using the side information $X_{\alpha }$ and $X_{\beta }$ are denoted by $C_{1}$ and $C_{2}$, respectively, and the overall code is denoted by $\left(C_{1},C_{2}\right)$. To propose formal design criteria to choose the subsets of P(X) \{ϕ} as queries, we define a singleton block in a query as the set of codewords that consist of only a single message bit. Similarly, we define an *n*-tuple sum block in a query, for 2≤n<K, as the set of codewords that consist of XOR bit additions of *n* different messages.

### 2.1. Design Criteria for XOR-Based PIR-PSI Codes

For N=2, M=2 and any K≥3, let $\left(C_{1},C_{2}\right)$ be an XOR-based code to retrieve $X_{\gamma }$ using the side information $X_{\alpha }$ and $X_{\beta }$ The code $\left(C_{1},C_{2}\right)$ is said to be XOR-based PIR-PSI code if keeping the query to $N_{1}$ (or $N_{2}$) unchanged, it is possible to design a query to $N_{2}$ (or $N_{1}$) such that

1. Condition 1: any demand can be retrieved from $N_{1}$ and $N_{2}$ using any two side information messages.
2. Condition 2: the structure of the new query to $N_{2}$ (or $N_{1}$) is same as that of $C_{2}$ (or $C_{1}$), namely: (i) the number of singleton and *n*-tuple sum blocks is the same, for any 2≤n≤K−1, and (ii) the frequency distribution of the message bits across all the codewords in the query is the same as that of $C_{2}$ (or $C_{1}$).

We take a two-step approach to design codes satisfying the above criteria. First, we present the queries of the code for a given demand $X_{\gamma }$ and a pair of side information $X_{\alpha }$ and $X_{\beta }$, and prove the correctness of the construction in retrieving $X_{\gamma }$. Subsequently, we present a rigorous proof to show that keeping the query to $N_{1}$ (or $N_{2}$) unchanged, new queries to $N_{2}$ (or $N_{1}$) can be constructed by satisfying Condition 1 and Condition 2. We show that the rate of code is more than that of [2], implying that our codes can be used when sequentially downloading files at different time-instants. The construction procedure for XOR-based PIR-PSI code is explained in the next section.

## 3. XOR-Based PIR Codes with Private Side Information

Among the *K* messages, let $X_{1}$ be the demand, and $X_{2}$ and $X_{3}$ be the side information. With M=2, our construction is applicable only for K≥3. For K=3, the code construction is trivial with rate one since an XOR version of all the files can be downloaded. For K>3, the ingredients and the instructions provided in the next two sections must be followed to obtain the queries for $N_{1}$ and $N_{2}$. Although the individual bits of the *K* files will be used to retrieve the *L* bits of $X_{1}$, first, our construction provides a way to place the file index in the query, and then describes a way to choose the specific bits of each file in the query. Along with the steps for ingredients and code construction, a running example for K=4, with messages A,B,C,D is also presented, wherein *A* plays the role of demand, i.e., $X_{1}$, *B* and *C* play the role of side information, i.e., $X_{2}$, and $X_{3}$.

### 3.1. Ingredients and Construction Strategy

With $X=\{X_{1},X_{2},\ldots ,X_{K-1}\}$, we construct P(X)={Φ(V)|∀V∈PS(X)}, where Φ(·) is as introduced in Definition 1, PS(X) is the power set of X. In the context of the running example with K=4, we have X={A,B,C}, and therefore, P({A,B,C}) is given in Table 6.

Owing to the use of power set, the elements of P(X) are unique, generating a total of $2^{K-1}$ elements using $2^{K-2}$ copies of each message. Towards converting P(X) into a query, we will allocate distinct indices for each copy of the message, therefore resulting in $2^{K-2}$ unique bits of a message. This will ensure that the query at this stage will contain K−1 messages each containing $L=2^{K-2}$ bits. In order to prepare the desired query with *K* messages, we need to add $2^{K-2}$ copies of the message $X_{K}$ to the existing elements of P(X). In the context of the example, as seen in Table 6, 4 bits of A,B,C are present in P({A,B,C}). Therefore, 4 copies of *D* should be added at different positions. In the next section, we provide a set of instructions to add $X_{K}$.

### 3.2. Algorithm for Code Construction

**1.** Rewrite $P(\left\{X_{1},X_{2},\ldots ,X_{K-1}\right\})$ as $P(\left\{X_{1},X_{2}\right\})$⨂$P(\left\{X_{3},X_{4},\cdots ,X_{K-1}\right\})$, where ⨂ operator can be defined on two sets $M_{1}$ and $M_{2}$ as

$$M_{1}⨂M_{2}=\left\{\alpha +\beta ,\;|\;\forall \;\alpha \in M_{1},\beta \in M_{2}\right\},$$

such that ϕ+ϕ=ϕ, α+ϕ=α and ϕ+β=β. For the example with K=4, it is straightforward to verify that applying ⨂ operator on P({A,B}) and P({C}), as given in Table 7, gives P({A,B,C}) given in Table 6.

**2.** From Step **1**, we know that the size of $P(\left\{X_{3},X_{4},\cdots ,X_{K-1}\right\})$ is $2^{K-3}$. Form a new table with two columns, namely: Column 1 and Column 2. Excluding ϕ, replicate the $2^{K-3}-1$ entries of $P(\left\{X_{3},X_{4},\cdots ,X_{K-1}\right\})$ into two columns, therefore forming a total of $2\left(2^{K-3}-1\right)=2^{K-2}-2$ elements. For the example, ϕ is removed from P({C}), and the remaining element is replicated into 2 columns as shown in Table 8.

**3.** Add $X_{K}$ to all the entries of Column 1. Leave Column 2 unaltered. Through this step, out of the $2^{K-2}$ copies, $2^{K-3}-1$ copies of $X_{K}$ are added. For the example, the two columns are as shown in Table 9.

**4.** Form a new table with two columns, namely: Column 1’ and Column 2’. Perform ⨂ operation between $\left\{\phi ,X_{1}+X_{2}\right\}$ and all the entries in the two-tuple sum block and three-tuple sum block of Column 1 from Step **3**, and place the result in Column 1’. Similarly, perform ⨂ operation between $\left\{X_{1},X_{2}\right\}$ and all the entries in the singleton block and two-tuple sum block of Column 2 from Step **3**, and place the result in Column 2’. In the example, only a singleton block is present, and therefore, the corresponding Column 1’ and Column 2’ are presented in Table 10.

**5.** Skip this step if K<=5 since largest value of *n* for the *n*-tuple sum block is 2. This is already addressed in the previous step.

- If *K* = 6 or 7, perform ⨂ operation between $\left\{X_{1},X_{2}\right\}$ and all the entries in the *n*-tuple sum block, for n∈{4,5,…,K−2} of Column 1, and append the result in Column 1’. Similarly, perform ⨂ operation between $\left\{\phi ,X_{1}+X_{2}\right\}$ and all the entries in the *n*-tuple sum block, for n∈{3,4,…,K−3}, of Column 2, and append the result in Column 2’.
- If K>7, perform ⨂ operation between $\left\{X_{1},X_{2}\right\}$ and all the entries in the *n*-tuple sum block, for n∈{4,5,…,K−3} of Column 1, and append the result in Column 1’. Similarly, perform ⨂ operation between $\left\{\phi ,X_{1}+X_{2}\right\}$ and all the entries in the *n*-tuple sum block, for n∈{3,4,…,K−4}, of Column 2, and append the result in Column 2’. However, for the (K−2)-tuple sum block in Column 1, perform ⨂ operation with $\left\{\phi ,X_{1}+X_{2}\right\}$ and append the result in Column 1’. Similarly, for the (K−3)-tuple sum block in Column 2, perform ⨂ operation $\left\{X_{1},X_{2}\right\}$ and append the result in Column 2’.

This step is not applicable to the running example since K=4. At the end of this step, both Column 1’ and Column 2’ contain $2^{K-2}-2$ elements each owing to the ⨂ operation. With this, we highlight that the union of Column 1’and Column 2’ has generated $2^{K-1}-4$ elements of $P(\left\{X_{1},X_{2},\ldots ,X_{K-1}\right\})$. This does not include $\left\{\phi ,X_{1},X_{2},X_{1}+X_{2}\right\}$ since ϕ of $P(\left\{X_{3},X_{4},\ldots ,X_{K-1}\right\})$ was excluded when constructing Column 1 and Column 2 in Step **2**. Furthermore, since $X_{K}$ was already added to Column 1 (containing $2^{K-3}-1$ elements), at the end of Step **5**, Column 1’ contains $2^{K-2}-2$ copies of $X_{K}$. This implies that only two more copies of $X_{K}$ are to be added to ensure that each message has $2^{K-2}$ copies. In the example, Step **3** added one copy of *D*, and Step **4** made it two copies. Now two more copies are remaining.

**6.** From $\left\{\phi ,X_{1},X_{2},X_{1}+X_{2}\right\}$, omit ϕ, and add $X_{K}$ to $X_{2}$. This generates $\left\{X_{1},X_{2}+X_{K},X_{1}+X_{2}\right\}$. Place these elements in a new column, referred to as Column 3. Please note that one more copy of $X_{K}$ must be added to achieve $2^{K-2}$ copies. In the example, Column 3 is given in Table 11.

**7.** Form the query to $N_{1}$ by taking the union of all the elements in Column 1’, Column 2’ and Column 3. By construction, this set contains $X_{1}+X_{3}$ coming from Column 2’. Therefore, add the last remaining copy of $X_{K}$ to $X_{1}+X_{3}$, and update it as $X_{1}+X_{3}+X_{K}$. Thus, we have a total of $2^{K-1}-1$ elements in this query constructed by adding $2^{K-2}$ copies of *K* files $\left\{X_{1},X_{2},\ldots ,X_{K}\right\}$. Finally, in the query to $N_{1}$, provide distinct indices to every copy of a message therefore ensuring that every bit of a message is used only once. Overall, the query is a set of linear combinations of $\left\{X_{i,j}\;|\;i\in \left[K\right],j\in \left[L\right]\right\}$. In the example, the query to $N_{1}$ after following the above steps are given in Table 12 (without indices) and Table 13 (with indices).

Before we present the procedure to construct the query to $N_{2}$, we present special structures in the query defined as *the known byproduct combination* and the *unknown byproduct combination*, and then present some results on their structure. Known byproduct combination are the bit combinations of byproducts in a codeword that does not contain the demand index. Formally, if $X_{1}$ is the demand, $X_{2},X_{3}$ are the side information, and the format of the codeword is H+W, where $H\in P(\left\{X_{2},X_{3}\right\})$ and $W\in P(\left\{X_{4},X_{5},\ldots ,X_{K}\right\})$\{ϕ}, then *W* is the known byproduct combination. Informally, this is the combination of byproduct messages that can be retrieved from the database. Unknown byproduct combinations are the bit combinations of the byproducts in a codeword that contains the demand index with or without the side information message bits. Formally, if $X_{1}$ is the demand, $X_{2},X_{3}$ are the side information, and the codeword is of the form $X_{1}+H+W$, where $H\in P(\left\{X_{2},X_{3}\right\})$ and $W\in P(\left\{X_{3},X_{4},\ldots ,X_{K}\right\})$ \{ϕ}, then *W* is the unknown byproduct combination. Informally, this is the combination of byproduct messages that cannot be retrieved from the database due to the unknown demand index that is along with it.

**Proposition** **1.** *If the message combination $W\in P(\left\{X_{4},X_{5},\ldots ,X_{K}\right\})$\{ϕ} appears as an unknown byproduct in the query to $N_{1}$, then it also exists as a known byproduct, but with different index values on each message.*

**Proof.**  By definition, if *W* is an unknown byproduct, then it appears in the query along with the demand $X_{1}$. Since *W* may appear alone or along with the side information messages, we shall denote the query associated with the unknown byproduct as $U=X_{1}+f\left(X_{2},X_{3}\right)+W$, where $f\left(X_{2},X_{3}\right)\in \left\{\phi ,X_{2},X_{3},X_{2}+X_{3}\right\}$. From the code construction, *U* must be equal to $X_{1}+X_{3}+X_{K}$ that was added in Step **7**, or it must belong to either Column 1’ or Column 2’ of Step **4** and Step **5**. If $U=X_{1}+X_{3}+X_{K}$, then the corresponding known byproduct is available in Column 3 of Step **6**. However, if *U* is available in Column 1’, then the corresponding known byproduct is also in Column 1’ because the elements of Column 1’ are generated by performing ⨂ operation with either $\left\{X_{1},X_{2}\right\}$ or $\left\{\phi ,X_{1}+X_{2}\right\}$. The same argument is also applicable if *U* is available in Column 2’. Finally, the index values used on the known byproduct are different from that of unknown byproduct since every copy of a message is assigned different indices as per Step **7**. This completes the proof.  □

**8.** To generate query to $N_{2}$, the following instructions must be followed. Copy the structure of $N_{1}$ as it is without the indices of bits. For the demand $X_{1}$, the first $2^{K-2}$ bits are already queried in $N_{1}$. Therefore, give the next $2^{K-2}$ numbers as the indices of $X_{1}$ in $N_{2}$. Use the same index number on each message of the side information as that of the query in $N_{1}$. From Proposition 1, the query to $N_{1}$ produces a symmetric sequence of known and unknown byproduct combinations. List the unknown and known byproduct combinations in two separate columns in the ascending order of *n*-tuple length following the lexicographical order and ascending order of indices of bits. For a given byproduct combination of messages in the unknown column, an identical byproduct combination exists in the known column, but with the difference that the index values used by the unknown combination is different from the known combination. To assign index values on the byproduct messages of $N_{2}$, for a given byproduct combination, use the index values of the unknown combination of $N_{1}$ on the known combination of $N_{2}$, and vice-versa. This ensures that all the byproduct messages can be indexed using one-to-one mapping between the unknown and the known column.

For the given example, in the fourth and fifth bit of Table 13, $D_{1}$ and $D_{2}$ are with side information, and therefore, they become the known byproduct bits. Similarly, in the sixth and seventh bit of the query to $N_{1}$, $D_{3}$ and $D_{4}$ are with the demand, and therefore, they become unknown byproduct bits, as listed in Table 14. To generate the query for $N_{2}$, the structure of $N_{1}$ is copied without the indices. The indices of the demand are {5,6,7,8} in $N_{2}$. The indices of B,C are maintained as they are. Based on the one-to-one mapping in Table 14, $D_{3}$ and $D_{1}$ are swapped, and so are $D_{4}$ and $D_{2}$. Finally, the query for $N_{2}$ is as shown in Table 15. This completes the code construction.

**Theorem** **1.** *For N=2 and any K>3, with the knowledge of side information $X_{2}$ and $X_{3}$, the proposed code construction can retrieve $2^{K-2}$ bits of $X_{1}$ per database by downloading $2^{K-1}-1$ bits per database. Thus, the rate of the code is*

(1) $$R=\frac{2^{K-2}}{2^{K-1}-1}.$$

**Proof.**  From the code construction, the query to $N_{1}$ and $N_{2}$ are obtained by adding $2^{K-2}$ bits of $X_{K}$ to the power set structure $P(\left\{X_{1},X_{2},\ldots ,X_{K-1}\right\})$\{ϕ}. Since the cardinality of $P(\left\{X_{1},X_{2},\ldots ,X_{K-1}\right\})$\{ϕ} is $2^{K-1}-1$, and every message in $P(\left\{X_{1},X_{2},\ldots ,X_{K-1}\right\})$\{ϕ} appears $2^{K-2}$ times, the rate of the code is as given in (1). In the rest of the proof, we show that every bit of $X_{1}$ can be retrieved from $N_{1}$ and $N_{2}$ using the side information. If a bit of $X_{1}$ appears in the form of $X_{1}+f\left(X_{2},X_{3}\right)$ either in $N_{1}$ or $N_{2}$, where $f\left(X_{2},X_{3}\right)\in \left\{\phi ,X_{2},X_{3},X_{2}+X_{3}\right\}$, then this bit can be retrieved since $X_{1}$ and $X_{2}$ are known. On the other hand, if a bit of $X_{1}$ appears in the form of $X_{1}+f\left(X_{2},X_{3}\right)+W$ on database $N_{1}$ (or $N_{2}$), where $W\in P(\left\{X_{4},X_{5},\ldots ,X_{K}\right\})$\{ϕ}, then from Step **8** of the code construction this bit can also be retrieved using the side information and the corresponding known byproduct of *W*, which is downloaded from $N_{2}$ (or $N_{1}$). This completes the proof.  □

The above theorem provided the proof for correctness to retrieve $X_{1}$ using $X_{2}$ and $X_{3}$ as the side information, for any K>3. In the next section, we show that the proposed codes satisfy the joint-privacy criteria in Section 2.1.

## 4. Proof for Joint Privacy of XOR-Based PIR-PSI Codes

Our approach is to use the code construction that is designed for the case when $X_{1}$ is the demand and $X_{2},X_{3}$ are the side information messages. Using this code, we fix the query submitted to $N_{1}$, and then show by construction that a query to $N_{2}$ can be generated in such a way that any demand can be retrieved from the two databases using any two messages as the side information. Considering the case of arbitrary values of *K*, constructions are provided in three parts, namely:

- By fixing $X_{K}$ as one of the side information messages, we show that a query to $N_{2}$ can be synthesized such that any two messages out of the remaining K−1 messages can play the role of the demand and the other side information.
- With $X_{K}$ as the demand, we show that a query to $N_{2}$ can be synthesized such that any two messages out of the remaining K−1 messages play the role of side information.
- By fixing $X_{K}$ as one of the byproducts, we show that a query to $N_{2}$ can be synthesized such that any three messages out of the remaining K−1 messages can play the role of the demand and two side information.

We start with the construction when $X_{K}$ is a side information. Since the code construction is presented by adding $2^{K-2}$ copies of $X_{K}$ to $P(\left\{X_{1},X_{2},\ldots ,X_{K-1}\right\})$\{ϕ}, the privacy proof for this case is directly dependent on the power set structure of $P(\left\{X_{1},X_{2},\ldots ,X_{K-1}\right\})$\{ϕ}. Given the power set structure, for any K−3 byproducts bits, the known and the unknown byproduct combinations in the query to $N_{1}$ are symmetric. As a result, the query submitted to $N_{2}$ must follow the same structure as that of $N_{1}$ except that (i) the index values of the demand will change from $2^{K-2}+1$ to $2^{K-1}$ instead of 1 to $2^{K-2}$, (ii) the known and unknown byproduct combinations are swapped similar to the proposed construction, and (iii) the index values of all the side information can be retained as they were in $N_{1}$. The method used for constructing query to $N_{2}$ under this case is summarized in the first row of Table 16.

When $X_{K}$ is the demand message, for any given combination of the side information, i.e., $X_{i}X_{j}$ such that i≠j and i,j∈[K]\{K}, we first list the corresponding known and unknown byproduct combinations using the existing query at $N_{1}$. Please note that these byproduct combinations will involve the bits of messages other than that of $X_{K},X_{i},X_{j}$. Subsequently, we pick the query submitted to $N_{1}$, and then apply a suitable transformation between $\left\{X_{1},X_{2},X_{3}\right\}$ and $\left\{X_{1},X_{2},\ldots ,X_{K-1}\right\}$ such that the known and the unknown byproduct combinations of the modified query are symmetric. Finally, if the known (or the unknown) byproduct combination in the query to $N_{1}$ is available as an unknown (or known) byproduct in the modified query, we swap their indices, otherwise, we perform suitable modifications to the modified query such that the demand $X_{K}$ can be retrieved. Given that the proposed code construction is applicable when $X_{1}$ is the demand and $X_{2},X_{3}$ are the side information, the same set of transformations is applicable on the query to $N_{1}$ for the side information messages within the following classes, namely: (i) the side information is one of $\left\{X_{1}X_{2},X_{2}X_{3}\right\}$, (ii) the side information is one of $\left\{X_{1}X_{i}\;|\;2\leq i\leq K-1,i\neq 3\right\}$, (iii) the side information is one of $\left\{X_{2}X_{i}\;|\;3\leq i\leq K-1\right\}$, (iv) the side information is one of $\left\{X_{3}X_{i}\;|\;4\leq i\leq K-1\right\}$, and (v) the side information is one of $\left\{X_{i}X_{j}\;|\;4\leq i,j\leq K-1,i\neq j\right\}$. For each of these cases, the set of transformations that must be carried on the query submitted to $N_{1}$ is presented in the third column of Table 16, whereas the set of manipulations that must be applied after the transformation is listed in the fourth column of Table 16. It is important to note that after applying the transformations in the third column, a subset of the unknown and known byproduct combinations of the query to $N_{1}$ would be symmetric with that of the transformed query. On those subsets, the known and unknown byproduct combinations must be swapped similar to the case when $X_{K}$ plays the role of a side information.

Finally, when $X_{K}$ is a byproduct, for a given demand and side information messages, we propose a sequence of transformations between the messages in the query to $N_{1}$ to ensure that the known and the unknown byproduct combinations match as much as that with that of the query to $N_{1}$. For the cases when the known and unknown byproduct combinations do not match, we propose modifications on the transformed query to retrieve all the bits of the demand. Table 17 lists the instructions to obtain the query at $N_{2}$ for the case when $X_{K}$ is one of the byproducts.

In both Table 16 and Table 17, the operator ⇌ represents swapping the elements on either side of the ⇌ operator, i.e., swap all the position of element in left hand side of ⇌ with the one in its right-hand side in the given query. For example, A⇌B implies that swap all positions of *A* and *B* in the given query. If the ⇌ operator has two juxtaposed elements in either side, swap first and second element of the left side with first and second element of the right side, respectively. Similarly, the operator ⇒ represents the replacement of the codeword in the left of the ⇒ operator with the one in the right. For example, A+B⇒C+D implies replacing the codeword A+B with C+D in the given query. In summary, the first row of Table 16, shows that when $X_{K}$ is a side information, any demand can be retrieved with any side information pair in $N_{2}$ while keeping the query at $N_{1}$ unaltered and making necessary changes in $N_{2}$ without altering the structure of code in $N_{1}$. Similarly, Table 16 and Table 17 show that the same is possible when $X_{K}$ is the demand and $X_{K}$ is one of the byproducts, respectively. This shows that the query obtained from the code construction algorithm in Section 3 can be used to retrieve any demand with any pair of side information in $N_{2}$ while keeping the query at $N_{1}$ unaltered.

Please note that Table 16 and Table 17 only provide the instructions to obtain the queries to $N_{2}$ for any demand-side information combination keeping the query at $N_{1}$ fixed. However, the correctness of the joint-privacy proof follows from the fact that the instructions for any particular demand-side information combination retraces back to the structure of query at $N_{1}$, which was proved to be correct in Theorem 1.

## 5. Proof for Joint Privacy for the Code with K=7

In this section, we demonstrate the joint-privacy proof using our code with K=7 as presented in Table 18. We choose K=7 for this demonstration since using K<7 will not cover all the cases mentioned in Table 16 and Table 17. On the other hand, using K>7 as an example is not feasible for exposition due to large queries, of length 127 and beyond. In this example, the letter *G* will play the role of $X_{K}$, whereas the letters A,B,C,…,F play the role of $X_{1},X_{2},\ldots ,X_{K-1}$, respectively. The objective of this section is to show the execution of instructions presented in Table 16 and Table 17.

### 5.1. G as a Side Information

Recall that the queries for K>3, M=2 is formed by adding $2^{K-2}$ copies of $X_{K}$ (in this case *G* for K=7) to $P(\left\{X_{1},X_{2},\ldots ,X_{K-1}\right\})$\{ϕ} (in this case the power set structure P({A,B,C,D,E,F})\{ϕ}). Since *G* is the side information, it can be removed from downloaded bits, and therefore, the privacy proof of the code is directly dependent on the structure of P({A,B,C,D,E,F})\{ϕ}. For any given demand and any side information from the remaining five messages, structure of P({A,B,C,D,E,F})\{ϕ} guarantees that the known byproduct combinations and the unknown byproduct combinations in the query to $N_{1}$ are symmetric. Hence, the query submitted to $N_{2}$ follows the same structure as that of $N_{1}$ except that

- The index values of the demand will change from 33 to 64 instead of 1 to 32.
- The known and unknown byproduct combinations are swapped similar to the proposed construction.
- The index values of all the side information can be retained as they were in $N_{1}$.

### 5.2. G as the Demand

When *G* is the demand, the two side information messages can come from {A,B,…,E,F} in K−12 = 62 = 15 ways. The 15 combinations are {AB,AC,AD,AE,AF,BC,BD,BE,BF,CD,CE,CF,DE,DF,EF}, implying that the two letters juxtaposed next to each other are the side information messages. These 15 combinations are grouped into five different types, namely: {AC,BC}, {AB,AD,AE,AF}, {BD,BE,BF}, {CD,CE,CF} and {DE,DF,EF}. The reason for this classification is attributed to the fact that the query to $N_{1}$ was constructed assuming *A* as the demand and B,C as the side information, and therefore, when we have to design the new query to $N_{2}$, it should match with the existing query to $N_{1}$.

#### 5.2.1. One of {AC,BC} as Side Information and *G* as the Demand

When either AC or BC are the side information messages, the known and unknown byproduct combinations are symmetric. With the side information AC, the pattern of unknown and known byproduct combinations without the indices are given in Table 19. Therefore, the demand *G* can be retrieved by swapping the indices of the known and unknown byproduct combinations. Similar to the code construction, when submitting the query to $N_{2}$, the indices on side information must be the same, whereas the indices of the demand on $N_{2}$ must be new.

In the case when BC are the side information messages, the pattern of the known and the unknown byproduct combinations remains symmetric, and therefore, the query to $N_{2}$ is similar to that when AC were the side information messages. The only exception is that the role of *A* and *B* is swapped as *A* becomes a byproduct and *B* becomes a side information. Thus, all the bits of *G* can be retrieved from both databases without altering the structure of query to $N_{1}$ when one of {AC,BC} are the side information messages.

#### 5.2.2. One of {AB,AD,AE,AF} as Side Information and *G* as the Demand

In the case when one of {AB,AD,AE,AF} are the side information, the known and unknown byproduct combinations are almost symmetric with one exception that a singleton bit of byproduct *C* goes to the unknown side from the known side. For instance, when AB are the side information, the known and unknown byproduct combinations are as shown in the first two columns of Table 20. Therefore, the query to $N_{2}$ should somehow accommodate this extra unknown bit without changing the structure. To construct a query to $N_{2}$, we use the query to $N_{1}$ and make appropriate modifications as discussed hereafter. For exposition, we take the case when AB are the side information. We already know that the query to $N_{1}$ gives a symmetric known-unknown byproduct combinations for side information AC and demand *G*. We pick the query submitted to $N_{1}$, and propose a simple transformation of *B*⇌*C*, i.e., swapping all positions of *B* with *C*, to generate a new query. Due to the transformation, it is clear that this new query gives a symmetric known-unknown byproduct combinations when *G* is the demand and AB are the side information. This pattern of unknown and known byproduct combinations are shown in the third and the fourth columns of Table 20.

We now provide modifications on the new query after the transformation *B*⇌*C* so that we can use as our query to $N_{2}$. To help this cause, the original query to $N_{1}$ for K=7 (without indices) is numbered from 1 to 63 for each codeword in Table 18. Since one bit of *C* moved to the unknown side from the known side in the existing query to $N_{1}$, we pick the new query (after the transformation) and then swap the singleton bit *A* (at bit number 1) and the bit *C* from the two-tuple sum C+G (bit number 10). This ensures that the extra unknown bit *C* is retrieved in bit number 1 while still being able to retrieve the demand *G* bit in bit number 10 since *A* is a side information. This sequence of operations from taking a copy of the query to $N_{1}$, the transformation of *B*⇌*C*, and the final exchange in bit positions 1 and 10 are displayed in the last three columns of Table 21. The rows of Table 21 indicated in red are the ones that are modified. Thus, the last column of this table forms the query to $N_{2}$ when AB are the side information. Of course, swapping of the known-unknown byproducts indices is required wherever they are symmetric.

In general, when handling the other cases of {AD,AE,AF} as the side information, the procedure for obtaining query for $N_{2}$ is similar to that when AB is the side information. We pick the query submitted to $N_{1}$ and perform the transformation of *C* with D,E,F for the cases AD,AE,AF, respectively. The modifications made to the new query after the transformation is the same as that when AB were the side information. Finally, the bit numbers would change according to the new position of codeword C+G.

#### 5.2.3. One of {BD,BE,BF} as Side Information and *G* as the Demand

In the case when one of {BD,BE,BF} are the side information, the known and unknown byproduct combinations are almost symmetric with two exceptions that a two-tuple sum of byproduct combination, A+C goes to the unknown side from the known side while a bit of byproduct *A* goes to the known side from the unknown side. For instance, when BD are the side information, the known and unknown byproduct combinations are as shown in the first two columns of Table 22. Therefore, the query to $N_{2}$ should somehow accommodate these deviations without changing the structure. To construct a query to $N_{2}$, we use the query to $N_{1}$ and make appropriate modifications as discussed hereafter. For exposition, we take the case when BD are the side information. We already know that the query to $N_{1}$ gives a symmetric known-unknown byproduct combinations for side information AC and demand *G*. We pick the query submitted to $N_{1}$, and perform a transformation of *A*⇌*B* and *C*⇌*D*, to generate a new query. Due to the transformation, it is clear that this new query gives a symmetric known-unknown byproduct combinations when *G* is the demand and BD are the side information. This pattern of unknown and known byproduct combinations are shown in the third and the fourth columns of Table 22.

Since a bit of A+C is moved to the unknown side from the known side in the existing query to $N_{1}$, there is only one known bit of A+C available to retrieve *G*. But the query after transformation uses 2 bits of A+C (bit number 39 and 45) to retrieve 2 bits of *G* as seen in the third column of Table 23. Since we have one extra bit of *A* available, to obtain the extra bit of A+C, the bit C+G in bit number 12 is replaced with bit B+G. *G* is still retrievable since *B* is side information. The bit of *C* freed from bit number 12 along with extra bit of *A* contributes to the second A+C bit. Since one more *B* is added to the query, the singleton bit of *B* is replaced with demand bit *G* as seen in bit number 1 in the table below. Since one extra demand bit is retrieved, the bit number 38 is changed from B+A+D+G to B+A+D+C so that the extra unknown bit of A+C is also acquired. Since one bit of *C* was removed in bit number 12, this change will bring back the uniform distribution of bits while keeping the structure of query at $N_{2}$ maintained as that of $N_{1}$. This sequence of operations from taking a copy of the query to $N_{1}$, the transformation of *A*⇌*B* and *C*⇌*D*, and the final exchange in bit positions 1, 12, and 38 are displayed in the last three columns of Table 23. Thus, the last column of this table forms the query to $N_{2}$ when BD are the side information. Of course, swapping of the known-unknown byproducts indices is required wherever they are symmetric.

In general, when handling the other cases of {BE,BF} as the side information, the procedure for obtaining query for $N_{2}$ is similar to that when BD were the side information. We pick the query submitted to $N_{1}$ and perform the transformation between *A* with *B*, and then between *C* and *E* (or *F*), for BE (or BF) as the side information. The modifications made to the new query after the transformation when BD were the side information can be reproduced to retrieve *G* from $N_{2}$.

#### 5.2.4. One of {CD,CE,CF} as Side Information and *G* as the Demand

In the case when one of {CD,CE,CF} are the side information, there is a drastic change in the known and unknown byproduct combinations compared to the previous cases when of *G* is the demand. For instance, when CD are the side information the singleton bits of byproducts *A* and *B* are unknown only one time whereas they are known for three times. The two-tuple sum block combinations of byproducts except A+B are unknown only one time but are known three times whereas A+B is unknown three times but known only one time. All the three-tuple sum blocks combinations of byproducts are unknown three times while they are known only once. The four-tuple sum block combinations of byproducts are known three times while they are unknown only once. The known and unknown byproduct combinations are as shown in Table 24.

The query to $N_{2}$ should somehow accommodate these deviations without changing the structure. To construct a query to $N_{2}$, we use the query to $N_{1}$ and make appropriate modifications as discussed hereafter. For exposition, we take the case when CD are the side information. We already know that the query to $N_{1}$ gives a symmetric known-unknown byproduct combinations for side information AC and demand *G*. We pick the query submitted to $N_{1}$, and perform the transformation *A*⇌*D*, to generate a new query. Due to the transformation, it is clear that this new query gives a symmetric known-unknown byproduct combinations when *G* is the demand and CD are the side information. Since one bit of A+B moves towards the unknown side from the known side, there is only one bit of A+B available in known bits to retrieve *G*. But the new query after transformation uses 2 bits of A+B (bit number 39 and 43) to retrieve 2 bits of *G* as seen in the third column of Table 25. This is rectified by swapping bit *G* in bit number 39 with *F*, therefore retrieving the extra bit of A+B+F required in the unknown while removing the necessity of second known bit of A+B. This increments and decrements the bit count (total number of bits of a message) of *F* and *G* respectively by 1. The extra bit of A+B required is obtained by swapping bit *E* in the bit number 19 with *B* since all the two-tuple sum combinations except A+B are unknown only once. This increments and decrements the bit count of *B* and *E* respectively by 1. Since the bit A+F is unknown only once, the bit number 29, which is the second query for A+F is changed to B+D+G to use the extra known bit *B* along with side information *D* to retrieve *G*. This neutralises the differences occurred in bit count of *F* and *G* and also increments the bit count of *B* and *D* and decrements the bit count of *A* and *C* respectively by 1. *B* has a net increment of 2 in the bit count. This increment of *D* and decrement of *C* is neutralised in bit number 56 by swapping *D* with *C*. Now the four-tuple sum bit A+B+E+F is known three times and is unknown only once. Therefore *B* and side information *C* are swapped in bit numbers 49 and 62. Bit number 49, which was initially querying a bit of A+B+E+F will now query the extra unknown bit of A+E+F. Bit number 62 was supposed to be the second query for *G* bit using A+E+F but all the three-tuple sum bits have only one known bit. This A+E+F is replaced by the extra known bit of A+B+E+F. The extra unknown bit of three-tuple sum bit B+E+F is retrieved by swapping *G* with *F* in bit number 40. This increments and decrements the bit count of *F* and *G* respectively by 1. Bit numbers 15 and 23 which queries the second bit *A* and B+E respectively are together used to retrieve the extra unknown bit of A+B+E since *A* and B+E are unknown only once. Bit numbers 57–59 uses three-tuple sums A+B+E,A+B+F and B+E+F to retrieve *G* for the second time but they are known only once. Therefore, these bits are obtained by combining the bits {A,B+E}, {A,B+F} and {B,E+F} since *A*, *B* and all the two-tuple sum blocks except A+B are known one extra time. B+D of bit number 38 is replaced with A+E and *B* of bit number 41 is replaced with *A* so that the extra two-tuple sum bits, A+E and A+F will be used to retrieve G. This neutralises the bit count of *B* and *E* while increments the bit count of *A* by 2 and decrements the bit count of *D* by 1. Since bit count of *A* was already lagging 1 bit behind, this step increases it by 2 bits to give a net increment of 1 for *A*. *C* of bit number 33 is replaced with *B* and C+A of bit number 28 is replaced with B+G so that the extra two-tuple sum bits, B+E and B+F will be used to retrieve *G*. This neutralises the bit count of *G* and *A* while decrements the bit count of *D* by 1. This also increments and decrements the bit count of *B* and *C* respectively by 2. The *B* from bit number 10 is swapped with side information *D*, while *D* itself in bit number 1 is swapped with *G*. The *G* bit and *B* bit of bit numbers 14 and 2 are swapped with *C* to obtain the second unknown bit of *F* and therefore neutralising B,C and *G*. *B* is swapped with *E* in both bit numbers 6 and 9 to retrieve extra unknown bit *E* and only unknown bit of E+F. This increments and decrements the bit count of *E* and *B* respectively by 2. *E* in bit numbers 21 and 31 are swapped with *G* and *B* respectively and *F* in bit number 31 is swapped with *D* to retrieve extra unknown bit of *F* and only unknown bit of *B*. This neutralises D,E and *F* while incrementing *B* and *G* one time with *B* with a net decrement of 1 bit. *G* of bit number 13 is swapped with *A* to retrieve the only known bit of A+E while *A* of bit number 30 is swapped with the last known bit of *B* to retrieve *G* which neutralises the bit count of *B* and *G* and achieving the uniform distribution of bits while keeping the structure of query at $N_{2}$ maintained as that of $N_{1}$. This sequence of operations from taking a copy of the query to $N_{1}$, the transformation of *A*⇌*D*, and the final exchange in bit positions are displayed in the last three columns of Table 25. Thus, the last column of this table forms the query to $N_{2}$ when CD are the side information. Of course, swapping of the known-unknown byproducts indices is required wherever they are symmetric.

In general, when handling the other cases of {CE,CF} as the side information, the procedure for obtaining query for $N_{2}$ is similar to that when CD are the side information. We pick the query submitted to $N_{1}$ and perform the transformation between *A* with *D*. The modifications made to the new query after transformation when CD were the side information can be reproduced to retrieve *G* from $N_{2}$.

#### 5.2.5. One of {DE,DF,EF} Is the Side Information and *G* Is the Demand

Compared to the previous case (Section 5.2.4), this case has one more singleton bit of *A* going to the known side from the unknown side and one bit of A+C going from the known side to the unknown side for all side information from {DE,DF,EF}. A minor modification to the previous code for $N_{2}$ seen in Table 25 can incorporate the change required. Since the fourth column of Table 25 gave the query to $N_{2}$ for demand *G* and side information CD, swap CD with the desired side information from {DE,DF,EF} to obtain a new query. Since one more *A* goes to the known side, all 4 bits of *A* are known. Therefore, a side information in the bit number 15 in the fourth column of Table 25 which queries the only unknown *A* can be replaced with *C* to make it retrieve A+C. Bit number 38 which retrieves *G* using A+C can be now used to retrieve *G* using the fourth known *A* bit by swapping *C* with the side information swapped before. Therefore, all bits of *G* can be retrieved from both databases when one of {DE,DF,EF} is the side information without altering the structure of query from $N_{1}$.

### 5.3. G as a Byproduct

G can be byproduct in (the demand can come from {A,B,C,D,E,F} in K−11 = 61 = 6) × (the 2 side information messages can come from {A,B,C,D,E,F}−Demand in K−22 = 52 = 10) = 6 × 10 = 60 ways. This is subdivided into 4 different cases.

#### 5.3.1. *A* Is the Demand

When *A* is the demand and BC are the side information, the known and unknown byproduct combinations are symmetric. The demand can be retrieved similar to the operation mentioned in step 8 of the code construction in Section 3. Now when one of {BD,BE,BF} are the side information there is a small difference from symmetry. In the unknown side one singleton bit of *C* and *G* is removed (does not go to the known side) and one bit of C+G is added to unknown (is not removed from known). The structure of code remains the same as that of $N_{1}$ since the *C* and *G* of the extra unknown C+G bit can be obtained from the individual querying itself since one singleton bit of *C* and *G* is removed from unknown. Now when one of {CD,CE,CF} are the side information, the known and unknown byproduct combinations are similar to the case when *G* was the demand and one of {CD,CE,CF} were the side information (Section 5.2.4). A simple transformation of *A*⇌*G* in the code structure provided in Section 5.2.4 will provide the necessary query to $N_{2}$. Now when one of {DE,DF,EF} is the side information, the known and unknown byproduct combinations are similar to the case of {CD,CE,CF} with a small difference. The difference is same as {BD,BE,BF} case, in the unknown side one singleton bit of *C* and *G* is removed (does not go to the known side) and one bit of C+G is added to unknown (is not removed from known). The structure of code remains the same as {CD,CE,CF} since the *C* and *G* of the extra unknown C+G bit can be obtained from the individual querying itself since one singleton bit of *C* and *G* is removed from unknown. This completes all side information cases for *A* as a demand and *G* as one of the byproducts. Therefore, all cases with *A* as a demand is retrievable.

#### 5.3.2. *B* Is the Demand

When *B* is the demand, the cases are similar to *A* as the demand except for the side information set {DE,DF,EF}. When *B* is the demand and AC are the side information, the known and unknown byproduct combinations are symmetric. The demand can be retrieved similar to the operation mentioned in step 8 of the code construction in Section 3. Now when one of {AD,AE,AF} are the side information there is a small difference from symmetry. In the unknown side one singleton bit of *C* and *G* is removed (does not go to the known side) and one bit of C+G is added to unknown (is not removed from known). The structure of code remains the same as that of $N_{1}$ since the *C* and *G* of the extra unknown C+G bit can be obtained from the individual querying itself since one singleton bit of *C* and *G* is removed from unknown. Now when one of {CD,CE,CF} are the side information, the known and unknown byproduct combinations are similar to the case when *G* was the demand and one of {CD,CE,CF} were the side information (Section 5.2.4). A simple transformation of *B*⇌*G* in the code structure provided in Section 5.2.4 will provide the necessary query to $N_{2}$. Now when one of {DE,DF,EF} is the side information, the known and unknown byproduct combinations are similar to the case when *G* was the demand and one of {DE,DF,EF} were the side information (Section 5.2.5) with a small difference. The unknown bit A+C is replaced with just *A* while the known bit A+G is replaced with A+C+G. When *G* was the demand and one of {CD,CE,CF} were the side information, for obtaining second known bit of A+B+E we had to combine individual bits of *A* and B+E as explained in Section 5.2.4. Similar operation was performed for A+B+C in Section 5.2.5. Since second A+C+G (which is analogous of A+B+C from Section 5.2.5) bit is available as known, we can use it directly to query demand *B* therefore freeing individual bits of *A* and C+G. Now this free known bit C+G is used in place of retrieving *B* with singleton *G*. This frees a singleton *G* and this along with the singleton *A* freed before is used to retrieve *B* with bit A+G (since the only known A+G was converted to A+C+G). Finally, the query bit that retrieves A+C is now used to retrieve the new unknown *A* bit. This completes all side information cases for *B* as a demand and *G* as one of the byproducts. Therefore, all cases with *B* as a demand is retrievable.

#### 5.3.3. *C* Is the Demand

When *C* is the demand and one of {AB,AD,AE,AF} are the side information, the known and unknown byproduct combinations are similar to the case when *G* was the demand and one of {AB,AD,AE,AF} were the side information (Section 5.2.2). The query for $N_{2}$ can be obtained similar to that case with a transformation of *C*⇌*G*. When one of {BD,BE,BF} are the side information, the known and unknown byproduct combinations are similar to the case when *G* was the demand and one of {BD,BE,BF} were the side information (Section 5.2.3). The query for $N_{2}$ can be obtained similar to that case with a transformation of *C*⇌*G*. When one of {DE,DF,EF} are the side information, the known and unknown byproduct combinations is similar to the one when *B* was the demand and one of {DE,DF,EF} were the side information (Section 5.3.2) with some minor differences. In the unknown side, one bit of A+G is removed while one bit of *A* and 2 bits of *G* are included. In the known side 2 bits of B+G are replaced by just *B* while one bit of A+B is replaced by A+B+G. For instance, when EF are the side information, the known and unknown byproduct combinations are as shown in the first two columns of Table 26.

The query to $N_{2}$ should somehow accommodate these deviations without changing the structure. For exposition, we take the case when EF are the side information. To construct a query to $N_{2}$, we use the query to $N_{2}$ from Table 25 and make appropriate modifications as discussed hereafter. To this query we perform the transformation CD⇌EF, to generate a new query. To this query we perform the modifications mentioned as in the case of *G* as the demand and one of {DE,DF,EF} as the side information to obtain a new query. To this query we perform a transformation *B*⇌*G* to obtain a new query. To this query we perform the modifications mentioned as in the case of *B* as the demand and one of {DE,DF,EF} as the side information to obtain a new query. Please note that this query is exactly the query to $N_{2}$ for the case of *B* as the demand and EF are the side information. To this query we perform a transformation of *B*⇌*C* as seen in third column of Table 27. Please note that the third column of Table 27 is not obtained by direct transformation of *B*⇌*C* in $N_{1}$. Rather, it is obtained after the execution of various steps as discussed earlier. Since one bit of A+G is removed from the unknown, bit number 7 which was initially querying A+G can now be used to query one extra bit of *G*. Bit number 2 which was just a combination of side information E+F can be used to query second extra bit of *G*. Bit number 8 and 28 are modified to query *C* with two extra known singleton *B* bits since one B+G bit in known set is removed. Since bit number 8 which queried B+G which was required to obtain third bit of unknown A+B+G is modified, the third bit of A+B+G is queried by modifying bit number 38. This modification removes the retrieval of one bit of demand using A+D. This is neutralised by modifying bit number 41 to remove query of demand with B+G (since 2 bits of B+G was removed from known set) and replace it with A+D. Since this A+B+G is queried directly, *A* retrieved in bit number 11 which was supposed to be the *A* in A+B+G can be now used to retrieve the extra singleton bit *A* which came to the unknown side. Finally, bit number 53 which was the bit that used A+B to query *C* can now be used to query A+B+D+G (since one bit of A+C is removed from known) while bit number 61 which was initially supposed to query A+B+D+G is now used to retrieve *C* with the extra A+B+G bit that is available in the known set. Query to $N_{1}$, the transformation of *B*⇌*C* to query of $N_{2}$ for the case of *B* as the demand and EF are the side information, and the final exchange in bit positions are displayed in the last three columns of Table 27. Thus, the last column of this table forms the query to $N_{2}$ when EF are the side information and *C* is the demand.

In general, when handling the other cases of {DE,DF} as the side information, the procedure for obtaining query for $N_{2}$ is similar to that when EF are the side information. We pick the query submitted to $N_{2}$ for the case of *B* as the demand and {DE,DF} as the side information respectively and perform the transformation between *B* with *C*. The modifications made to the new query after transformation when EF were the side information can be reproduced to retrieve *B* from $N_{2}$.

#### 5.3.4. One of {D,E,F} Is the Demand

When one of {D,E,F} is the demand, the known and unknown byproduct combinations are symmetric if either *A* or *B* is one of the side information. The demand can be retrieved similar to the operation mentioned in step 8 of the code construction in Section 3. For other side information pairs which does not have either *A* or *B* in side information, the known and unknown byproduct combinations are same as the previous case where *C* was the demand and one of {DE,DF,EF} were side information with two exceptions. One bit of *A* goes to the unknown side while one bit of A+G comes to the known side. For exposition let us consider *D* as the demand. To obtain the query for $N_{2}$, perform the transformation of *C*⇌*D* to the query for $N_{2}$ in Table 27. To this new query, use any bit that queries A+G to query *A*. Use any bit that retrieves *D* using *A* to retrieve *D* using A+G.

In general, when handling the other cases of {E,F} as the demand, pick the query submitted to $N_{2}$ for the case of *C* as the demand and {DE,DF,EF} as the side information respectively and perform the transformation between *C* with demand. The modifications made to the new query after transformation when *D* was the demand can be reproduced to retrieve demand from $N_{2}$. This completes all side information cases when one of {D,E,F} is the demand and *G* is one of the byproducts. Therefore, all cases with one of {D,E,F} as the demand are retrievable.

## 6. Discussion and Directions for Future Work

In this work, we have presented the first XOR-based code construction for a PIR-PSI setting involving N=2 databases and M=2 side information. We have shown that our codes achieve the rate $\frac{2^{K-2}}{2^{K-1}-1}$. Although our code construction marginally falls short of the capacity of PIR-PSI setting, it offers substantial reduction in the decoding complexity when compared to the MDS counterpart (which achieves the capacity). This implies that our codes are applicable when multiple files have to be downloaded by a user at different time-instants from the two databases. We believe that this work can be extended in one of the following directions, namely: (i) How to construct XOR-based PSR-PSI codes for N=2 non-colluding databases with arbitrary values of *M* side information messages? (ii) How to construct XOR-based PSR-PSI codes for arbitrary values of *N* and *M*? and finally, (iii) Do XOR-based PIR-PSI codes exist that achieve the capacity of the PSR-PSI setting? Importantly, we have shown that our codes provide rates strictly higher than the capacity of PIR schemes. Among the above set of questions posed for future research, we believe that solving (iii) is challenging. A probable reason for the fall in rate for XOR-based code constructions is the XOR constraint itself. In the simplest setting of K=4, the capacity is $\frac{2}{3}$. To achieve this rate, the query must consist of 3 codewords satisfying the criteria in Section 2.1 such that 2 of them retrieve the demand. Using exhaustive search, we observe that this scenario is hard to begin with, and therefore, we believe that the problem is challenging for arbitrary values of *K*.

## Figures and Tables

**Table 1:** Table comparing the rate of our scheme with the capacity of PIR-PSI setting.

Scheme	Rate	Method	Computational Complexity
[15]	$\frac{2^{K-3}}{2^{K-2}-1}$	MDS coding	High. Exponential as
	(higher than our code)		*K* increases. Finite field
			multiplication involved.
Our scheme	$\frac{2^{K-2}}{2^{K-1}-1}$	XOR	Low. Bit-wise XOR involved
[2]	$\frac{2^{K-1}}{2^{K}-1}$	XOR	Low. Bit-wise XOR involved
	(lower than our code)		

**Table 2:** Query obtained as per the proposed code construction when *A* is the demand, and *B* and *C* are the side information.

Query to $N_{1}$	Query to $N_{2}$
$A_{1}$	$A_{5}$
$A_{2}+B_{1}$	$A_{6}+B_{1}$
$B_{2}+C_{1}$	$B_{2}+C_{1}$
$B_{3}+D_{1}$	$B_{3}+D_{3}$
$C_{2}+D_{2}$	$C_{2}+D_{4}$
$A_{3}+C_{3}+D_{3}$	$A_{7}+C_{3}+D_{1}$
$A_{4}+B_{4}+C_{4}+D_{4}$	$A_{8}+B_{4}+C_{4}+D_{2}$

**Table 3:** For a given query to $N_{1}$ (in the first column), all possible queries to $N_{2}$ when *D* is a side information.

Query to $N_{1}$	(A,BD,C)	(A,CD,B)	(B,AD,C)	(B,CD,A)	(C,AD,B)	(C,BD,A)
$A_{1}$	$A_{5}$	$A_{5}$	$A_{1}$	$A_{2}$	$A_{1}$	$A_{3}$
$A_{2}+B_{1}$	$A_{6}+B_{1}$	$A_{6}+B_{2}$	$A_{2}+B_{5}$	$A_{1}+B_{5}$	$A_{2}+B_{2}$	$A_{4}+B_{1}$
$B_{2}+C_{1}$	$B_{2}+C_{3}$	$B_{1}+C_{3}$	$B_{6}+C_{2}$	$B_{6}+C_{2}$	$B_{1}+C_{5}$	$B_{2}+C_{5}$
$B_{3}+D_{1}$	$B_{3}+D_{1}$	$B_{4}+D_{1}$	$B_{7}+D_{1}$	$B_{7}+D_{1}$	$B_{4}+D_{1}$	$B_{4}+D_{1}$
$C_{2}+D_{2}$	$C_{4}+D_{2}$	$C_{4}+D_{2}$	$C_{1}+D_{2}$	$C_{1}+D_{2}$	$C_{6}+D_{2}$	$C_{6}+D_{2}$
$A_{3}+C_{3}+D_{3}$	$A_{7}+C_{1}+D_{3}$	$A_{7}+C_{1}+D_{3}$	$A_{3}+C_{4}+D_{3}$	$A_{4}+C_{4}+D_{3}$	$A_{3}+C_{7}+D_{3}$	$A_{1}+C_{7}+D_{3}$
$A_{4}+B_{4}+C_{4}+D_{4}$	$A_{8}+B_{4}+C_{2}+D_{4}$	$A_{8}+B_{3}+C_{2}+D_{4}$	$A_{4}+B_{8}+C_{3}+D_{4}$	$A_{3}+B_{8}+C_{3}+D_{4}$	$A_{4}+B_{3}+C_{8}+D_{4}$	$A_{2}+B_{3}+C_{8}+D_{4}$

**Table 4:** All possible queries to $N_{2}$ when *D* is a byproduct.

Query to $N_{1}$	(A,BC,D)	(B,AC,D)	(C,AB,D)
$A_{1}$	$A_{5}$	$A_{1}$	$D_{2}$
$A_{2}+B_{1}$	$A_{6}+B_{1}$	$A_{2}+B_{5}$	$A_{1}+D_{3}$
$B_{2}+C_{1}$	$B_{2}+C_{1}$	$B_{6}+C_{1}$	$B_{1}+C_{5}$
$B_{3}+D_{1}$	$B_{3}+D_{3}$	$B_{7}+D_{2}$	$B_{2}+D_{4}$
$C_{2}+D_{2}$	$C_{2}+D_{4}$	$C_{2}+D_{1}$	$A_{2}+C_{6}$
$A_{3}+C_{3}+D_{3}$	$A_{7}+C_{3}+D_{1}$	$A_{3}+C_{3}+D_{4}$	$A_{3}+B_{3}+C_{7}$
$A_{4}+B_{4}+C_{4}+D_{4}$	$A_{8}+B_{4}+C_{4}+D_{2}$	$A_{4}+B_{8}+C_{4}+D_{3}$	$A_{4}+B_{4}+C_{8}+D_{1}$

**Table 5:** All possible queries to $N_{2}$ when *D* is the demand.

Query to $N_{1}$	(D,BC,A)	(D,AC,B)	(D,AB,C)
$A_{1}$	$D_{5}$	$A_{1}$	$C_{2}$
$A_{2}+B_{1}$	$D_{6}+B_{1}$	$A_{2}+D_{5}$	$A_{1}+C_{3}$
$B_{2}+C_{1}$	$B_{2}+C_{1}$	$D_{6}+C_{1}$	$B_{1}+D_{5}$
$B_{3}+D_{1}$	$B_{3}+A_{3}$	$D_{7}+B_{2}$	$B_{2}+C_{4}$
$C_{2}+D_{2}$	$C_{2}+A_{4}$	$C_{2}+B_{3}$	$A_{2}+D_{6}$
$A_{3}+C_{3}+D_{3}$	$D_{7}+C_{3}+A_{1}$	$A_{3}+C_{3}+B_{4}$	$A_{3}+B_{3}+D_{7}$
$A_{4}+B_{4}+C_{4}+D_{4}$	$D_{8}+B_{4}+C_{4}+A_{2}$	$A_{4}+D_{8}+C_{4}+B_{2}$	$A_{4}+B_{4}+D_{8}+C_{1}$

**Table 6:** P(X) for the running example.

P({A,B,C})
ϕ
A
B
C
A+B
A+C
B+C
A+B+C

**Table 7:** Step 1 applied on the running example.

P({A,B})	P({C})
ϕ	ϕ
A	C
B	
A+B	

**Table 8:** Step 2 applied on the running example.

Column 1	Column 2
C	C

**Table 9:** Step 3 applied on the running example.

Column 1	Column 2
C+D	C

**Table 10:** Step 4 applied on the running example.

Column 1’	Column 2’
C+D	C+A
C+D+A+B	C+B

**Table 11:** Step 6 applied on the running example.

Column 3
A
B+D
A+B

**Table 12:** Step 7 applied on the running example without indices.

Query to $N_{1}$ without Indices
*A*
A+B
B+C
B+D
C+D
A+C+D
A+B+C+D

**Table 13:** Step 7 applied on the running example with indices.

Query to $N_{1}$ with Indices
$A_{1}$
$A_{2}+B_{1}$
$B_{2}+C_{1}$
$B_{3}+D_{1}$
$C_{2}+D_{2}$
$A_{3}+C_{3}+D_{3}$
$A_{4}+B_{4}+C_{4}+D_{4}$

**Table 14:** Unknown and known byproducts of the running example.

Unknown	Known
$D_{3}$	$D_{1}$
$D_{4}$	$D_{2}$

**Table 15:** Final query to $N_{1}$ and $N_{2}$.

Query to $N_{1}$	Query to $N_{2}$
$A_{1}$	$A_{5}$
$A_{2}+B_{1}$	$A_{6}+B_{1}$
$B_{2}+C_{1}$	$B_{2}+C_{1}$
$B_{3}+D_{1}$	$B_{3}+D_{3}$
$C_{2}+D_{2}$	$C_{2}+D_{4}$
$A_{3}+C_{3}+D_{3}$	$A_{7}+C_{3}+D_{1}$
$A_{4}+B_{4}+C_{4}+D_{4}$	$A_{8}+B_{4}+C_{4}+D_{2}$

**Table 16:** Instructions to obtain query at $N_{2}$ when $X_{K}$ is either SI or demand.

No.	Case	Transformation	Manipulations
1	$X_{K}$ as SI	None	Swap the known and unknown
			byproducts to obtain query at $N_{2}$.
2	$X_{K}$ as demand,	None	Swap the known and unknown
	$X_{1}X_{3}$ or $X_{2}X_{3}$ are SI		byproducts to obtain query at $N_{2}$.
3	$X_{K}$ as demand	Pick query at	Swap the singleton bit $X_{1}$
	$X_{1}X_{i}$ are SI	$N_{1}$ and apply	and the bit $X_{3}$ from
	2≤i≤K−1, i≠3	$X_{i}⇌X_{3}$	the two-tuple sum $X_{3}+X_{K}$
4	$X_{K}$ as demand	Pick query at	$X_{3}+X_{K}$⇒$X_{2}+X_{K}$
	$X_{2}X_{i}$ are SI	$N_{1}$ and apply	$X_{2}$⇒$X_{K}$
	4≤i≤K−1	$X_{1}X_{3}$⇌$X_{2}X_{i}$	$X_{2}+X_{1}+X_{i}+X_{K}$⇒$X_{2}+X_{1}+X_{i}+X_{3}$
5	$X_{K}$ as demand	Pick query at	If i=4 and K≤7, perform the following:
	$X_{3}X_{i}$ are SI	$N_{1}$ and apply	$X_{4}$⇒$X_{K}$, $X_{2}+X_{4}$⇒$X_{3}+X_{4}$, $X_{2}+X_{3}$⇒$X_{5}+X_{3}$,
	4≤i≤K−1	$X_{1}$⇒$X_{4}$	$X_{2}+X_{6}$⇒$X_{5}+X_{6}$, $X_{2}+X_{K}$⇒$X_{4}+X_{K}$,$X_{5}+X_{K}$⇒
			$X_{5}+X_{1}$, $X_{6}+X_{K}$⇒$X_{6}+X_{3}$, $X_{4}+X_{1}+X_{5}$⇒
			$X_{4}+X_{1}+X_{2}$, $X_{4}+X_{5}+X_{6}$⇒$X_{4}+X_{K}+X_{6}$,
			$X_{3}+X_{1}+X_{5}$⇒$X_{2}+X_{K}+X_{5}$, $X_{3}+X_{1}+X_{6}$⇒
			$X_{2}+X_{K}+X_{4}$, $X_{3}+X_{1}+X_{K}$⇒$X_{2}+X_{K}+X_{3}$,
			$X_{3}+X_{5}+X_{6}$⇒$X_{3}+X_{2}+X_{4}$, $X_{3}+X_{6}+X_{K}$⇒
			$X_{2}+X_{6}+X_{K}$, $X_{4}+X_{2}+X_{3}+X_{K}$⇒$X_{1}+X_{5}+X_{3}+X_{K}$,
			$X_{4}+X_{2}+X_{1}+X_{K}$⇒$X_{1}+X_{2}+X_{6}+X_{4}$,
			$X_{4}+X_{2}+X_{5}+X_{K}$⇒$X_{4}+X_{2}+X_{5}+X_{6}$,
			$X_{4}+X_{2}+X_{6}+X_{K}$⇒$X_{4}+X_{1}+X_{6}+X_{K}$,
			$X_{4}+X_{2}+X_{1}+X_{5}+X_{6}$⇒$X_{4}+X_{3}+X_{1}+X_{5}+X_{6}$,
			$X_{4}+X_{1}+X_{5}+X_{6}+X_{K}$⇒$X_{3}+X_{1}+X_{5}+X_{6}+X_{K}$,
			$X_{4}+X_{3}+X_{1}+X_{5}+X_{6}+X_{K}$⇒
			$X_{4}+X_{2}+X_{1}+X_{5}+X_{6}+X_{K}$. For i≠4 and K≤7
			apply $X_{4}⇌X_{i}$ to the query obtained after transformation
			in column 3 and perform the manipulations mentioned above.
			For K>7, rearrange the query in column 3 such a way that
			the first $2^{K-2}-1$ codewords do not contain $X_{t}$ where
			t=max({4,5,…,K−1}),t≠i. Perform manipulations on
			these codewords by the steps proposed under the same
			scenario, but by treating the message set as
			$\left\{X_{1},X_{2},\ldots ,X_{K}\right\}$ \$\left\{X_{t}\right\}$. For the remaining $2^{K-2}$ codewords
			perform the following: Perform manipulations on all codewords
			of the form $X_{t}+Q+X_{K}$, where
			$Q\in P(\left\{X_{1},X_{2},\ldots ,X_{K-1}\right\}$ \$\left\{X_{t}\}\right)$ \{ϕ} similar to the steps
			proposed under the same scenario of K−1 messages,
			i.e., perform the same manipulations done to the codewords of
			the form $X_{t-1}+Q^{\prime }+X_{K-1}$, where
			$Q^{\prime }\in P(\left\{X_{1},X_{2},\ldots ,X_{K-2}\right\}$ \$\left\{X_{t-1}\}\right)$ \{ϕ} when number of
			messages was K−1. Similarly, the manipulations performed
			for the codewords of the form $X_{t-1}+Q^{\prime }$ when number of
			messages was K−1 should be repeated for all codewords of
			the form $X_{t}+Q$. If K=8, perform the following (else skip):
			$X_{1}+X_{m}+X_{n}+X_{o}+U⇌$$X_{m}+X_{n}+X_{o}+U$ and
			$X_{2}+X_{m}+X_{n}+X_{o}+U$$⇌X_{1}+X_{2}+X_{m}+X_{n}+X_{o}+U$,
			where U∈ $P(\left\{X_{3},X_{i}\right\})$, 4≤m,n,o≤K−1,m≠n≠o≠i.
6	$X_{K}$ as demand	Pick query at	$X_{p}+X_{q}+X_{1}$⇒$X_{q}+X_{3}+X_{1}$
	$X_{p}X_{q}$ are SI	$N_{2}$ obtained	$X_{1}+X_{t}+X_{3}+X_{K}$⇒$X_{1}+X_{t}+X_{p}+X_{K}$
	4≤p,q≤K−1	from case 5	where $X_{t}$ is some byproduct
	p≠q	$X_{3}X_{i}⇌X_{p}X_{q}$	other than $X_{1}$ and $X_{3}$.

**Table 17:** Instructions to obtain query at $N_{2}$ when $X_{K}$ one of the byproducts.

No.	Case	Transformation	Manipulations
9	$X_{1}$ as demand, $X_{2}X_{i}$	None	Swap the known and unknown
	are SI, 3≤i≤K−1		byproducts to obtain query at $N_{2}$.
10	$X_{1}$ as demand, $X_{p}X_{q}$	Pick query at	None
	are SI, 3≤p,q≤K−1	$N_{2}$ obtained	
	p≠q	from case 5	
		$X_{3}X_{i}⇌X_{p}X_{q}$	
		$X_{1}⇌X_{K}$	
11	$X_{2}$ as demand, $X_{1}X_{i}$	None	Swap the known and unknown
	are SI, 3≤i≤K−1		byproducts to obtain query at $N_{2}$.
12	$X_{2}$ as demand, $X_{p}X_{q}$	Pick query at	None
	are SI, 3≤p,q≤K−1	$N_{2}$ obtained	
	p≠q	from case 5	
		$X_{3}X_{i}⇌X_{p}X_{q}$	
		$X_{2}⇌X_{K}$	
13	$X_{3}$ as demand	Pick query at	None
	$X_{1}X_{i}$ are SI	$N_{2}$ obtained	
	2≤i≤K−1, i≠3	from Case 3	
		$X_{3}⇌X_{K}$	
14	$X_{3}$ as demand	Pick query at	None
	$X_{2}X_{i}$ are SI	$N_{2}$ obtained	
	4≤i≤K−1, i≠3	from Case 4	
		$X_{3}⇌X_{K}$	
15	$X_{3}$ as demand	Pick query at	$X_{i}+X_{j}\Rightarrow$$X_{K}+X_{j}$, $X_{K}+X_{1}\Rightarrow$$X_{K}+X_{i}$, $X_{K}+X_{2}\Rightarrow$
	$X_{i}X_{j}$ are SI	$N_{2}$ obtained	$X_{3}+X_{2}$, $X_{K}+X_{3}+X_{2}\Rightarrow$$X_{j}+X_{3}+X_{2}$,
	4≤i,j≤K−1, i≠j	from Case 10	$X_{2}+X_{K}+X_{j}+X_{3}\Rightarrow X_{1}+X_{4}+X_{i}+X_{3}$,
		$X_{2}⇌X_{3}$	$X_{j}+X_{1}+X_{4}+X_{3}\Rightarrow X_{j}+X_{1}+X_{2}+X_{K}$
			$X_{j}+X_{i}+X_{1}+X_{2}+X_{3}\Rightarrow X_{1}+X_{K}+X_{2}+X_{4}+X_{j}$
			$X_{j}+X_{K}+X_{i}+X_{1}+X_{2}+X_{4}\Rightarrow$
			$X_{1}+X_{2}+X_{K}+X_{3}+X_{i}+X_{j}$
16	$X_{\delta }$ as demand, $X_{i}X_{j}$	None	Swap the known and unknown
	are SI, 4≤δ≤K−1		byproducts to obtain query at $N_{2}$.
	1≤i≤2,2≤j≤K−1,		
	i≠j,i,j≠δ		
17	$X_{\delta }$ as demand, $X_{p}X_{q}$	Pick query at	None
	are SI, 4≤δ≤K−1,	$N_{2}$ obtained	
	3≤p,q≤K−1, p≠q	from case 5	
	p,q≠δ	$X_{3}X_{i}⇌X_{p}X_{q}$	
		$X_{\delta }⇌X_{K}$	
18	$X_{\delta }$ as demand, $X_{p}X_{q}$	Pick query at	$X_{1}+X_{\delta }\Rightarrow X_{2}+X_{\delta }$, $X_{2}+X_{\delta }+X_{p}\Rightarrow X_{1}+X_{K}+X_{\delta }$,
	are SI, 4≤δ≤K−1,	$N_{2}$ obtained	$X_{1}+X_{K}+X_{q}\Rightarrow X_{1}+X_{q}+X_{p}$
	4≤p,q≤K−1, p≠q	from case 5	
	p,q≠δ	$X_{3}X_{i}⇌X_{p}X_{q}$	
		$X_{\delta }⇌X_{K}$	

**Table 18:** Query to $N_{1}$ and $N_{2}$ as per our code construction.

Query to $N_{1}$	Query to $N_{2}$
$A_{1}$	$A_{33}$
$A_{2}+B_{1}$	$A_{34}+B_{1}$
$A_{3}+D_{1}$	$A_{35}+D_{2}$
$A_{4}+E_{1}$	$A_{36}+E_{2}$
$A_{5}+F_{1}$	$A_{37}+F_{2}$
$B_{2}+C_{1}$	$B_{2}+C_{1}$
$B_{3}+D_{2}$	$B_{3}+D_{1}$
$B_{4}+E_{2}$	$B_{4}+E_{1}$
$B_{5}+F_{2}$	$B_{5}+F_{1}$
$B_{6}+G_{1}$	$B_{6}+G_{6}$
$C_{2}+G_{2}$	$C_{2}+G_{13}$
$D_{3}+G_{3}$	$D_{16}+G_{14}$
$E_{3}+G_{4}$	$E_{16}+G_{15}$
$F_{3}+G_{5}$	$F_{16}+G_{16}$
$A_{6}+C_{3}+D_{4}$	$A_{38}+C_{3}+D_{7}$
$A_{7}+C_{4}+E_{4}$	$A_{39}+C_{4}+E_{7}$
$A_{8}+C_{5}+F_{4}$	$A_{40}+C_{5}+F_{7}$
$A_{9}+C_{6}+G_{6}$	$A_{41}+C_{6}+G_{1}$
$A_{10}+D_{5}+E_{5}$	$A_{42}+D_{8}+E_{8}$
$A_{11}+D_{6}+F_{5}$	$A_{43}+D_{9}+F_{8}$
$A_{12}+E_{6}+F_{6}$	$A_{44}+E_{9}+F_{9}$
$B_{7}+C_{7}+D_{7}$	$B_{7}+C_{7}+D_{4}$
$B_{8}+C_{8}+E_{7}$	$B_{8}+C_{8}+E_{4}$
$B_{9}+C_{9}+F_{7}$	$B_{9}+C_{9}+F_{4}$
$B_{10}+D_{8}+E_{8}$	$B_{10}+D_{5}+E_{5}$
$B_{11}+D_{9}+F_{8}$	$B_{11}+D_{6}+F_{5}$
$B_{12}+E_{9}+F_{9}$	$B_{12}+E_{6}+F_{6}$
$C_{10}+D_{10}+E_{10}$	$C_{10}+D_{18}+E_{18}$
$C_{11}+D_{11}+F_{10}$	$C_{11}+D_{19}+F_{18}$
$C_{12}+D_{12}+G_{7}$	$C_{12}+D_{20}+G_{17}$
$C_{13}+E_{11}+F_{11}$	$C_{13}+E_{19}+F_{19}$
$C_{14}+E_{12}+G_{8}$	$C_{14}+E_{20}+G_{18}$
$C_{15}+F_{12}+G_{9}$	$C_{15}+F_{20}+G_{19}$
$D_{13}+E_{13}+F_{13}$	$D_{21}+E_{21}+F_{21}$
$D_{14}+E_{14}+G_{10}$	$D_{22}+E_{22}+G_{20}$
$D_{15}+F_{14}+G_{11}$	$D_{23}+F_{22}+G_{21}$
$E_{15}+F_{15}+G_{12}$	$E_{23}+F_{23}+G_{22}$
$A_{13}+B_{13}+C_{16}+G_{13}$	$A_{45}+B_{13}+C_{16}+G_{2}$
$A_{14}+B_{14}+D_{16}+G_{14}$	$A_{46}+B_{14}+D_{3}+G_{3}$
$A_{15}+B_{15}+E_{16}+G_{15}$	$A_{47}+B_{15}+E_{3}+G_{4}$
$A_{16}+B_{16}+F_{16}+G_{16}$	$A_{48}+B_{16}+F_{3}+G_{5}$
$C_{17}+D_{17}+E_{17}+F_{17}$	$C_{17}+D_{30}+E_{30}+F_{30}$
$A_{17}+B_{17}+C_{18}+D_{18}+E_{18}$	$A_{49}+B_{17}+C_{18}+D_{10}+E_{10}$
$A_{18}+B_{18}+C_{19}+D_{19}+F_{18}$	$A_{50}+B_{18}+C_{19}+D_{11}+F_{10}$
$A_{19}+B_{19}+C_{20}+D_{20}+G_{17}$	$A_{51}+B_{19}+C_{20}+D_{12}+G_{7}$
$A_{20}+B_{20}+C_{21}+E_{19}+F_{19}$	$A_{52}+B_{20}+C_{21}+E_{11}+F_{11}$
$A_{21}+B_{21}+C_{22}+E_{20}+G_{18}$	$A_{53}+B_{21}+C_{22}+E_{12}+G_{8}$
$A_{22}+B_{22}+C_{23}+F_{20}+G_{19}$	$A_{54}+B_{22}+C_{23}+F_{12}+G_{9}$
$A_{23}+B_{23}+D_{21}+E_{21}+F_{21}$	$A_{55}+B_{23}+D_{13}+E_{13}+F_{13}$
$A_{24}+B_{24}+D_{22}+E_{22}+G_{20}$	$A_{56}+B_{24}+D_{14}+E_{14}+G_{10}$
$A_{25}+B_{25}+D_{23}+F_{22}+G_{21}$	$A_{57}+B_{25}+D_{15}+F_{14}+G_{11}$
$A_{26}+B_{26}+E_{23}+F_{23}+G_{22}$	$A_{58}+B_{26}+E_{15}+F_{15}+G_{12}$
$A_{28}+C_{25}+D_{25}+F_{24}+G_{24}$	$A_{60}+C_{25}+D_{28}+F_{27}+G_{28}$
$A_{29}+C_{26}+E_{25}+F_{25}+G_{25}$	$A_{61}+C_{26}+E_{28}+F_{28}+G_{29}$
$A_{30}+D_{26}+E_{26}+F_{26}+G_{26}$	$A_{62}+D_{29}+E_{29}+F_{29}+G_{30}$
$B_{27}+C_{27}+D_{27}+E_{27}+G_{27}$	$B_{27}+C_{27}+D_{24}+E_{24}+G_{23}$
$B_{28}+C_{28}+D_{28}+F_{27}+G_{28}$	$B_{28}+C_{28}+D_{25}+F_{24}+G_{24}$
$B_{29}+C_{29}+E_{28}+F_{28}+G_{29}$	$B_{29}+C_{29}+E_{25}+F_{25}+G_{25}$
$B_{30}+D_{26}+E_{26}+F_{26}+G_{26}$	$B_{30}+D_{29}+E_{29}+F_{29}+G_{30}$
$A_{31}+B_{31}+C_{30}+D_{30}+E_{30}+F_{30}$	$A_{63}+B_{31}+C_{30}+D_{17}+E_{17}+F_{17}$
$A_{32}+C_{31}+D_{31}+E_{31}+F_{31}+G_{31}$	$A_{64}+C_{31}+D_{32}+E_{32}+F_{32}+G_{32}$
$B_{32}+C_{32}+D_{32}+E_{32}+F_{32}+G_{32}$	$B_{32}+C_{32}+D_{31}+E_{31}+F_{31}+G_{31}$

**Table 19:** Pattern of unknown-known byproducts for SI AC.

Unknown	Known
B	B
B	B
D	D
D	D
E	E
E	E
F	F
F	F
B+D	B+D
B+D	B+D
B+E	B+E
B+E	B+E
B+F	B+F
B+F	B+F
D+E	D+E
D+E	D+E
D+F	D+F
D+F	D+F
E+F	E+F
E+F	E+F
B+D+E	B+D+E
B+D+E	B+D+E
B+D+F	B+D+F
B+D+F	B+D+F
B+E+F	B+E+F
B+E+F	B+E+F
D+E+F	D+E+F
D+E+F	D+E+F
B+D+E+F	B+D+E+F
B+D+E+F	B+D+E+F

**Table 20:** Pattern of unknown-known byproducts for SI AB and after *B*⇌*C*.

Unknown	Known	Unknown FOR *B* ⇌ *C*	Known FOR *B* ⇌ *C*
C			
C		C	C
C	C	C	C
D	D	D	D
D	D	D	D
E	E	E	E
E	E	E	E
F	F	F	F
F	F	F	F
C+D	C+D	C+D	C+D
C+D	C+D	C+D	C+D
C+E	C+E	C+E	C+E
C+E	C+E	C+E	C+E
C+F	C+F	C+F	C+F
C+F	C+F	C+F	C+F
D+E	D+E	D+E	D+E
D+E	D+E	D+E	D+E
D+F	D+F	D+F	D+F
D+F	D+F	D+F	D+F
E+F	E+F	E+F	E+F
E+F	E+F	E+F	E+F
C+D+E	C+D+E	C+D+E	C+D+E
C+D+E	C+D+E	C+D+E	C+D+E
C+D+F	C+D+F	C+D+F	C+D+F
C+D+F	C+D+F	C+D+F	C+D+F
C+E+F	C+E+F	C+E+F	C+E+F
C+E+F	C+E+F	C+E+F	C+E+F
D+E+F	D+E+F	D+E+F	D+E+F
D+E+F	D+E+F	D+E+F	D+E+F
C+D+E+F	C+D+E+F	C+D+E+F	C+D+E+F
C+D+E+F	C+D+E+F	C+D+E+F	C+D+E+F

**Table 21:** Final query to $N_{2}$ given in the fourth column.

Bit Number	$N_{1}$	*B*⇌*C*	$N_{2}$
1	A	A	C
2	A+B	A+C	A+C
3	A+D	A+D	A+D
4	A+E	A+E	A+E
5	A+F	A+F	A+F
6	B+C	C+B	C+B
7	B+D	C+D	C+D
8	B+E	C+E	C+E
9	B+F	C+F	C+F
10	B+G	C+G	A+G
11	C+G	B+G	B+G
12	D+G	D+G	D+G
13	E+G	E+G	E+G
14	F+G	F+G	F+G
15	A+C+D	A+B+D	A+B+D
16	A+C+E	A+B+E	A+B+E
17	A+C+F	A+B+F	A+B+F
18	A+C+G	A+B+G	A+B+G
19	A+D+E	A+D+E	A+D+E
20	A+D+F	A+D+F	A+D+F
21	A+E+F	A+E+F	A+E+F
22	B+C+D	C+B+D	C+B+D
23	B+C+E	C+B+E	C+B+E
24	B+C+F	C+B+F	C+B+F
25	B+D+E	C+D+E	C+D+E
26	B+D+F	C+D+F	C+D+F
27	B+E+F	C+E+F	C+E+F
28	C+D+E	B+D+E	B+D+E
29	C+D+F	B+D+F	B+D+F
30	C+D+G	B+D+G	B+D+G
31	C+E+F	B+E+F	B+E+F
32	C+E+G	B+E+G	B+E+G
33	C+F+G	B+F+G	B+F+G
34	D+E+F	D+E+F	D+E+F
35	D+E+G	D+E+G	D+E+G
36	D+F+G	D+F+G	D+F+G
37	E+F+G	E+F+G	E+F+G
38	A+B+C+G	A+C+B+G	A+C+B+G
39	A+B+D+G	A+C+D+G	A+C+D+G
40	A+B+E+G	A+C+E+G	A+C+E+G
41	A+B+F+G	A+C+F+G	A+C+F+G
42	C+D+E+F	B+D+E+F	B+D+E+F
43	A+B+C+D+E	A+C+B+D+E	A+C+B+D+E
44	A+B+C+D+F	A+C+B+D+F	A+C+B+D+F
45	A+B+C+D+G	A+C+B+D+G	A+C+B+D+G
46	A+B+C+E+F	A+C+B+E+F	A+C+B+E+F
47	A+B+C+E+G	A+C+B+E+G	A+C+B+E+G
48	A+B+C+F+G	A+C+B+F+G	A+C+B+F+G
49	A+B+D+E+F	A+C+D+E+F	A+C+D+E+F
50	A+B+D+E+G	A+C+D+E+G	A+C+D+E+G
52	A+B+E+F+G	A+C+E+F+G	A+C+E+F+G
53	A+C+D+E+G	A+B+D+E+G	A+B+D+E+G
54	A+C+D+F+G	A+B+D+F+G	A+B+D+F+G
55	A+C+E+F+G	A+B+E+F+G	A+B+E+F+G
56	A+D+E+F+G	A+D+E+F+G	A+D+E+F+G
57	B+C+D+E+G	C+B+D+E+G	C+B+D+E+G
58	B+C+D+F+G	C+B+D+F+G	C+B+D+F+G
59	B+C+E+F+G	C+B+E+F+G	C+B+E+F+G
60	B+D+E+F+G	C+D+E+F+G	C+D+E+F+G
61	A+B+C+D+E+F	A+C+B+D+E+F	A+C+B+D+E+F
62	A+C+D+E+F+G	A+B+D+E+F+G	A+B+D+E+F+G
63	B+C+D+E+F+G	C+B+D+E+F+G	C+B+D+E+F+G

**Table 22:** Pattern of unknown and known byproducts for SI BD and after *A*⇌*B* and *C*⇌*D*.

Unknown	Known	Unknown FOR *A* ⇌ *B* C⇌D	Known FOR *A* ⇌ *B* C⇌D
	A		
	A	A	A
A	A	A	A
C	C	C	C
C	C	C	C
E	E	E	E
E	E	E	E
F	F	F	F
F	F	F	F
A+C	A+C		
A+C		A+C	A+C
A+C		A+C	A+C
A+E	A+E	A+E	A+E
A+E	A+E	A+E	A+E
A+F	A+F	A+F	A+F
A+F	A+F	A+F	A+F
C+E	C+E	C+E	C+E
C+E	C+E	C+E	C+E
C+F	C+F	C+F	C+F
C+F	C+F	C+F	C+F
E+F	E+F	E+F	E+F
E+F	E+F	E+F	E+F
A+C+E	A+C+E	A+C+E	A+C+E
A+C+E	A+C+E	A+C+E	A+C+E
A+C+F	A+C+F	A+C+F	A+C+F
A+C+F	A+C+F	A+C+F	A+C+F
A+E+F	A+E+F	A+E+F	A+E+F
A+E+F	A+E+F	A+E+F	A+E+F
C+E+F	C+E+F	C+E+F	C+E+F
C+E+F	C+E+F	C+E+F	C+E+F
A+C+E+F	A+C+E+F	A+C+E+F	A+C+E+F
A+C+E+F	A+C+E+F	A+C+E+F	A+C+E+F

**Table 23:** Final query to $N_{2}$ given in the fourth column.

Bit Number	$N_{1}$	*A*⇌*B*, *C*⇌*D*	$N_{2}$
1	A	B	G
2	A+B	B+A	B+A
3	A+D	B+C	B+C
4	A+E	B+E	B+E
5	A+F	B+F	B+F
6	B+C	A+D	A+D
7	B+D	A+C	A+C
8	B+E	A+E	A+E
9	B+F	A+F	A+F
10	B+G	A+G	A+G
11	C+G	D+G	D+G
12	D+G	C+G	B+G
14	F+G	F+G	F+G
15	A+C+D	B+D+C	B+D+C
16	A+C+E	B+D+E	B+D+E
17	A+C+F	B+D+F	B+D+F
18	A+C+G	B+D+G	B+D+G
19	A+D+E	B+C+E	B+C+E
20	A+D+F	B+C+F	B+C+F
21	A+E+F	B+E+F	B+E+F
22	B+C+D	A+D+C	A+D+C
23	B+C+E	A+D+E	A+D+E
24	B+C+F	A+D+F	A+D+F
25	B+D+E	A+C+E	A+C+E
26	B+D+F	A+C+F	A+C+F
27	B+E+F	A+E+F	A+E+F
28	C+D+E	D+C+E	D+C+E
29	C+D+F	D+C+F	D+C+F
30	C+D+G	D+C+G	D+C+G
31	C+E+F	D+E+F	D+E+F
32	C+E+G	D+E+G	D+E+G
33	C+F+G	D+F+G	D+F+G
34	D+E+F	C+E+F	C+E+F
35	D+E+G	C+E+G	C+E+G
36	D+F+G	C+F+G	C+F+G
37	E+F+G	E+F+G	E+F+G
38	A+B+C+G	B+A+D+G	B+A+D+C
39	A+B+D+G	B+A+C+G	B+A+C+G
40	A+B+E+G	B+A+E+G	B+A+E+G
41	A+B+F+G	B+A+F+G	B+A+F+G
42	C+D+E+F	D+C+E+F	D+C+E+F
43	A+B+C+D+E	B+A+D+C+E	B+A+D+C+E
44	A+B+C+D+F	B+A+D+C+F	B+A+D+C+F
45	A+B+C+D+G	B+A+D+C+G	B+A+D+C+G
46	A+B+C+E+F	B+A+D+E+F	B+A+D+E+F
47	A+B+C+E+G	B+A+D+E+G	B+A+D+E+G
48	A+B+C+F+G	B+A+D+F+G	B+A+D+F+G
49	A+B+D+E+F	B+A+C+E+F	B+A+C+E+F
50	A+B+D+E+G	B+A+C+E+G	B+A+C+E+G
51	A+B+D+F+G	B+A+C+F+G	B+A+C+F+G
52	A+B+E+F+G	B+A+E+F+G	B+A+E+F+G
53	A+C+D+E+G	B+D+C+E+G	B+D+C+E+G
54	A+C+D+F+G	B+D+C+F+G	B+D+C+F+G
55	A+C+E+F+G	B+D+E+F+G	B+D+E+F+G
56	A+D+E+F+G	B+C+E+F+G	B+C+E+F+G
57	B+C+D+E+G	A+D+C+E+G	A+D+C+E+G
58	B+C+D+F+G	A+D+C+F+G	A+D+C+F+G
59	B+C+E+F+G	A+D+E+F+G	A+D+E+F+G
60	B+D+E+F+G	A+C+E+F+G	A+C+E+F+G
61	A+B+C+D+E+F	B+A+D+C+E+F	B+A+D+C+E+F
62	A+C+D+E+F+G	B+D+C+E+F+G	B+D+C+E+F+G
63	B+C+D+E+F+G	A+D+C+E+F+G	A+D+C+E+F+G

**Table 24:** Pattern of unknown and known byproducts for SI CD.

Unknown	Known
A	A
B	A
E	A
E	B
E	B
F	B
F	E
F	F
A+B	A+B
A+B	A+E
A+B	A+E
A+E	A+E
A+F	A+F
B+E	A+F
B+F	A+F
E+F	B+E
A+B+E	B+E
A+B+E	B+E
A+B+E	B+F
A+B+F	B+F
A+B+F	B+F
A+B+F	E+F
A+E+F	E+F
A+E+F	E+F
A+E+F	A+B+E
B+E+F	A+B+F
B+E+F	A+E+F
B+E+F	B+E+F
A+B+E+F	A+B+E+F
	A+B+E+F
	A+B+E+F

**Table 25:** Final query to $N_{2}$ given in the fourth column.

Bit Number	$N_{1}$	*A*⇌*D*	$N_{2}$
1	A	D	G
2	A+B	D+B	D+C
3	A+D	D+A	D+A
4	A+E	D+E	D+E
5	A+F	D+F	D+F
6	B+C	B+C	E+C
7	B+D	B+A	B+A
8	B+E	B+E	B+E
9	B+F	B+F	E+F
10	B+G	B+G	D+G
11	C+G	C+G	C+G
12	D+G	A+G	A+G
13	E+G	E+G	E+A
14	F+G	F+G	F+C
15	A+C+D	D+C+A	D+C+A
16	A+C+E	D+C+E	D+C+E
17	A+C+F	D+C+F	D+C+F
18	A+C+G	D+C+G	D+C+G
19	A+D+E	D+A+E	D+A+B
20	A+D+F	D+A+F	D+A+F
21	A+E+F	D+E+F	D+G+F
22	B+C+D	B+C+A	B+C+A
23	B+C+E	B+C+E	B+C+E
24	B+C+F	B+C+F	B+C+F
25	B+D+E	B+A+E	B+A+E
26	B+D+F	B+D+F	B+D+F
27	B+E+F	B+E+F	B+E+F
28	C+D+E	C+A+E	B+G+E
29	C+D+F	C+A+F	B+D+G
30	C+D+G	C+A+G	C+B+G
31	C+E+F	C+E+F	C+B+D
32	C+E+G	C+E+G	C+E+G
33	C+F+G	C+F+G	B+F+G
34	D+E+F	A+E+F	A+E+F
35	D+E+G	A+E+G	A+E+G
36	D+F+G	A+F+G	A+F+G
37	E+F+G	E+F+G	E+F+G
38	A+B+C+G	D+B+C+G	A+E+C+G
39	A+B+D+G	D+B+A+G	A+B+F+D
40	A+B+E+G	D+B+E+G	D+B+E+F
41	A+B+F+G	D+B+F+G	D+A+F+G
42	C+D+E+F	C+A+E+F	C+A+E+F
43	A+B+C+D+E	D+B+C+A+E	D+B+C+A+E
44	A+B+C+D+F	D+B+C+A+F	D+B+C+A+F
45	A+B+C+D+G	D+B+C+A+G	D+B+C+A+G
46	A+B+C+E+F	D+B+C+E+F	D+B+C+E+F
47	A+B+C+E+G	D+B+C+E+G	D+B+C+E+G
48	A+B+C+F+G	D+B+C+F+G	D+B+C+F+G
49	A+B+D+E+F	D+B+A+E+F	D+C+A+E+F
51	A+B+D+F+G	D+B+A+F+G	D+B+A+F+G
52	A+B+E+F+G	D+B+E+F+G	D+B+E+F+G
53	A+C+D+E+G	D+C+A+E+G	D+C+A+E+G
54	A+C+D+F+G	D+C+A+F+G	D+C+A+F+G
55	A+C+E+F+G	D+C+E+F+G	D+C+E+F+G
56	A+D+E+F+G	D+A+E+F+G	C+A+E+F+G
57	B+C+D+E+G	B+C+A+E+G	B+C+A+E+G
58	B+C+D+F+G	B+C+A+F+G	B+C+A+F+G
59	B+C+E+F+G	B+C+E+F+G	B+C+E+F+G
60	B+D+E+F+G	B+A+E+F+G	B+A+E+F+G
61	A+B+C+D+E+F	D+B+C+A+E+F	D+B+C+A+E+F
62	A+C+D+E+F+G	D+C+A+E+F+G	D+B+A+E+F+G
63	B+C+D+E+F+G	B+C+A+E+F+G	B+C+A+E+F+G

**Table 26:** Pattern of unknown and known byproducts for SI EF.

Unknown	Known
A	A
A	A
B	A
B	A
B	B
D	B
D	B
D	G
G	G
G	G
G	D
A+G	A+B
A+G	B+G
B+G	D+G
A+B	D+G
D+G	D+G
A+D	A+D
B+D	A+D
A+B+G	A+D
A+B+G	B+D
A+B+G	B+D
A+D+G	B+D
A+D+G	A+B+G
A+D+G	A+B+G
B+D+G	A+B+G
B+D+G	A+D+G
B+D+G	B+D+G
A+B+D	A+B+D
A+B+D	A+B+D+G
A+B+D	A+B+D+G
A+B+D+G	A+B+D+G

**Table 27:** Final query to $N_{2}$ given in the fourth column.

Bit Number	$N_{1}$	*B*⇌*C*	$N_{2}$
1	A	C	C
2	A+B	F+E	F+G
3	A+D	F+A	F+A
4	A+E	F+B	F+B
5	A+F	F+D	F+D
6	B+C	B+E	B+E
7	B+D	G+A	G+E
8	B+E	G+B	C+B
9	B+F	B+D	B+D
10	B+G	F+C	F+C
11	C+G	E+C	E+C
12	D+G	A+C	A+C
13	E+G	E+A	E+A
14	F+G	D+E	D+E
15	A+C+D	E+B+A	E+B+A
16	A+C+E	F+E+B	F+E+B
17	A+C+F	F+E+D	F+E+D
18	A+C+G	F+E+C	F+E+C
19	A+D+E	F+A+G	F+A+G
20	A+D+F	F+A+D	F+A+D
21	A+E+F	F+C+D	F+C+D
22	B+C+D	G+E+A	G+E+A
23	B+C+E	G+E+B	G+E+B
24	B+C+F	G+E+D	G+E+D
25	B+D+E	G+A+B	G+A+B
26	B+D+F	G+F+D	G+F+D
27	B+E+F	G+B+D	G+B+D
28	C+D+E	G+C+B	F+C+B
29	C+D+F	G+F+C	G+F+C
30	C+D+G	E+G+C	E+G+C
31	C+E+F	E+G+F	E+G+F
32	C+E+G	E+B+C	E+B+C
33	C+F+G	G+D+C	G+D+C
34	D+E+F	A+B+D	A+B+D
35	D+E+G	A+B+C	A+B+C
37	E+F+G	B+D+C	B+D+C
38	A+B+C+G	B+G+F+C	A+D+E+C
39	A+B+D+G	A+G+D+F	A+G+D+F
40	A+B+E+G	F+G+B+D	F+G+B+D
41	A+B+F+G	F+A+D+C	F+A+B+G
42	C+D+E+F	E+A+B+D	E+A+B+D
43	A+B+C+D+E	F+G+E+A+B	F+G+E+A+B
44	A+B+C+D+F	F+G+E+A+D	F+G+E+A+D
45	A+B+C+D+G	F+G+E+A+C	F+G+E+A+C
46	A+B+C+E+F	F+G+E+B+D	F+G+E+B+D
47	A+B+C+E+G	F+G+E+B+C	F+G+E+B+C
48	A+B+C+F+G	F+G+E+D+C	F+G+E+D+C
49	A+B+D+E+F	F+E+A+B+D	F+E+A+B+D
50	A+B+D+E+G	F+G+A+B+C	F+G+A+B+C
51	A+B+D+F+G	F+G+A+D+C	F+G+A+D+C
52	A+B+E+F+G	F+G+B+D+C	F+G+B+D+C
53	A+C+D+E+G	F+E+A+B+C	A+G+B+D+F
54	A+C+D+F+G	F+E+A+D+C	F+E+A+D+C
55	A+C+E+F+G	F+E+B+D+C	F+E+B+D+C
56	A+D+E+F+G	E+A+B+D+C	E+A+B+D+C
57	B+C+D+E+G	G+E+A+B+C	G+E+A+B+C
58	B+C+D+F+G	G+E+A+D+C	G+E+A+D+C
59	B+C+E+F+G	G+E+B+D+C	G+E+B+D+C
60	B+D+E+F+G	G+A+B+D+C	G+A+B+D+C
61	A+B+C+D+E+F	F+G+E+A+B+D	A+B+G+C+E+F
62	A+C+D+E+F+G	F+G+A+B+D+C	F+G+A+B+D+C
63	B+C+D+E+F+G	G+E+A+B+D+C	G+E+A+B+D+C
